# Molecular mechanisms of the preventable causes of cancer in the United States

**DOI:** 10.1101/gad.314849.118

**Published:** 2018-07-01

**Authors:** Erica A. Golemis, Paul Scheet, Tim N. Beck, Eward M. Scolnick, David J. Hunter, Ernest Hawk, Nancy Hopkins

**Affiliations:** 1 Program in Molecular Therapeutics, Fox Chase Cancer Center, Philadelphia, Pennsylvania 19111, USA;; 2 Department of Epidemiology, University of Texas M.D. Anderson Cancer Center, Houston, Texas 77030, USA;; 3 Molecular and Cell Biology and Genetics Program, Drexel University College of Medicine, Philadelphia, Pennsylvania 19129, USA;; 4 Eli and Edythe L. Broad Institute of the Massachusetts Institute of Technology and Harvard University, Cambridge, Massachusetts 02142, USA;; 5 Nuffield Department of Population Health, University of Oxford, Medical Sciences Division, Oxford OX3 7LF, United Kingdom;; 6 Division of Cancer Prevention and Population Sciences, University of Texas M.D. Anderson Cancer Center, Houston Texas 77030, USA;; 7 Koch Institute for Integrative Cancer Research, Biology Department, Massachusetts Institute of Technology, Cambridge, Massachusetts 02139, USA

**Keywords:** cancer mechanisms, cancer prevention, early detection

## Abstract

Annually, there are 1.6 million new cases of cancer and nearly 600,000 cancer deaths in the United States alone. The public health burden associated with these numbers has motivated enormous research efforts into understanding the root causes of cancer. These efforts have led to the recognition that between 40% and 45% of cancers are associated with preventable risk factors and, importantly, have identified specific molecular mechanisms by which these exposures modify human physiology to induce or promote cancer. The increasingly refined knowledge of these mechanisms, which we summarize here, emphasizes the need for greater efforts toward primary cancer prevention through mitigation of modifiable risk factors. It also suggests exploitable avenues for improved secondary prevention (which includes the development of therapeutics designed for cancer interception and enhanced techniques for noninvasive screening and early detection) based on detailed knowledge of early neoplastic pathobiology. Such efforts would complement the current emphasis on the development of therapeutic approaches to treat established cancers and are likely to result in far greater gains in reducing morbidity and mortality.

## Abstract (precis)

The enormous public health burden associated with cancer motivates research into understanding the root causes of cancer. This review by Golemis et al. discusses preventable risk factors and specific molecular mechanisms by which these factors modify human physiology to induce or promote cancer and emphasizes the need for greater efforts toward primary and secondary cancer prevention.

Prevention and early detection, along with continual improvements in cancer treatment, have contributed substantially to declining U.S. cancer death rates. Less widely known is that proven methods of prevention and early detection could further reduce the incidence of adult cancers in the U.S. by at least a third to a half and reduce cancer deaths by ≥50%. Furthermore, while it is widely known that smoking causes lung cancer and that sunlight causes skin cancer, only a small fraction of the public is aware that smoking increases the incidence of cancer in more than a dozen sites or that viruses cause essentially all cervical cancers, all head and neck cancers (HNCs) not caused by smoking, and most liver cancers not caused by obesity or hepatotoxins (such as chronic alcohol use). While the long latency between cancer-initiating insult and diagnosable disease means that measurable decreases in cancer incidence would take some time to manifest (e.g., see Pirie et al. 2013), nevertheless, over the next two decades, a highly significant reduction of mortality could be observed with widespread adoption of prevention strategies.

One purpose of writing this review for basic scientists is to draw greater attention to the field of cancer prevention by noting the impact of prevention on cancer outcomes at the individual and population levels to date as well as its potential for the future. Our hope is that a concise but detailed summary of current knowledge of the mechanisms of action of known carcinogens and preventable causes of cancer may stimulate further research, leading to the identification of additional preventable causes of cancer and also identification of mechanism-based interventions to prevent carcinogens from causing cancer. We view such a review as timely, as spectacular advances in understanding the genetic and cellular basis of cancer have shed light on these issues and could lead to promising new research directions in prevention and early detection. There is a need for more basic scientists to work in this area, and we hope that this review will motivate such efforts.

Typically, the development of cancer in adults is the result of multiple mutations in many genes involved in controlling the growth of cells and altered metabolic changes in tumor cells and the tumor microenvironment that facilitate or accelerate the ultimate growth of the cancer (Hanahan and Weinberg 2011; Pickup et al. 2014). Examples of these principles are shown by examining the incidence of human cancers, many of which increase dramatically with age. For instance, the incidence of cancer of the large intestine increases by a factor of ∼1000 between the ages 30 and 80 (Cairns 1978). How can we explain this? Very early studies of carcinogenesis in animals began to yield insights into understanding the biology behind this observation. The studies showed a long lag between the application of a carcinogen (a chemical that causes cancer, commonly by introducing a change in DNA) and the actual detection of the cancer (Yamagiwa and Ichikawa 1918). These studies also demonstrated a second crucial principle: the cooperativity of such “initiator” agents that mutate DNA and “promoter” agents that act after the mutational event, often to stimulate the growth of initiated cells (Filler et al. 2007).

Such observations led to the proposal in the 1950s that cancer is the end result of a series of events within a single cell (Fisher and Hollomon 1951; Nording 1953; Armitage and Doll 1954; Fisher 1958). Mathematical modeling suggested that as many as six events might be needed to produce many human adult cancers, such as colon cancer (Armitage and Doll 1957; for discussion, see Cairns 1978). Today, decades of research in animal models and humans support the multistep model of carcinogenesis and reveal many of the changes and processes involved (Hanahan and Weinberg 2011). Changes in specific genes—i.e., oncogenes (which drive the growth of cells) or tumor suppressor genes (which retard the abnormal growth of cells)—are followed by stimulated growth to expand the population of initiated cells. As this expansion occurs, the potential for additional events within an initiated cell increases until that cell acquires the ability to escape growth controls, avoid the normal mechanisms of programmed cell death, escape immune surveillance, remodel a microenvironment in which to thrive, and metastasize. The changes in oncogenes or tumor suppressor genes may be changes affecting their DNA sequence or epigenetic changes that control the expression of these genes.

For most human cancers, it is not possible to practically infer how many independent events are needed to produce all of the changes that result in cancer. However, it is clear that the events, including both mutational changes and promotion, can be caused by either intrinsic mechanisms or extrinsic events (Hanahan and Weinberg 2011). As an intrinsic mechanism, mutations can be caused by errors in DNA replication followed by mistakes in repair. Alternatively, the mutational changes can be extrinsic, (i.e., caused by external carcinogens that are mutagenic). Further complicating attribution of mutations is that some intrinsically produced potential mutagens, such as reactive oxygen species (ROS) (Sabharwal and Schumacker 2014), are produced at damaging rates in response to preventable causes, such as obesity. Once either source of mutations occurs, intrinsic or extrinsic changes in cellular biochemistry can accelerate the growth of the initiated mutagenized cells and of cancer cells by various metabolic mechanisms. In addition to the now widely accepted multistep hypothesis, it is likely that aging itself also promotes cancer through systemic changes associated with the aging process (Campisi 2013; Aunan et al. 2017; Palmer et al. 2018).

With the current knowledge, prevention becomes more effective if one can identify the extrinsic causes of cancer and remove them or understand and either retard or block intrinsic causes of cancer. Early detection when cancers are minimally symptomatic (or screening, which relates to the even earlier detection of presymptomatic cancers or cancer precursors) can work for some types of cancer because of the long time frame over which many cancers develop and the fact that at least some cancers remain sufficiently contained so that, when first detected, they can be completely excised (Alam and Ratner 2001). Another approach—called “interception”—proposes to use knowledge of the events that give rise to cancers to treat the disease at earlier stages, including even before a cancer can be detected (Blackburn 2011).

We begin this review with a discussion of “primary prevention,” which we refer to simply as “prevention.” Primary prevention means preventing people from getting cancer in the first place by eliminating or reducing carcinogenic exposures; for example, by smoking cessation or vaccination. We review the environmental and behavioral carcinogens currently known to be responsible for a large fraction of U.S. cancer deaths, their mechanisms of action, and successes to date in eliminating exposure to these agents. We then review prospects for preventing cancer by drugs or other mechanism-based interventions. We examine the importance of identifying high-risk individuals who can most benefit from prevention or early detection. We then briefly turn to early detection itself and discuss the significant problems associated with population screening. We review successful screening methods in use in the U.S. and mechanistic reasons for their success. We conclude with the prospect for novel screening methods based on advances in the genetics and molecular biology of cancer. In the final section, we consider how much cancer is biologically intrinsic versus how much is caused by exposure to environmental, occupational, and behavioral carcinogens and the role of aging in cancer and the possibility of preventing or delaying cancer by delaying aging itself.

## The prevention of cancer

### Surprising discoveries from cancer epidemiology

#### In theory, the majority of cancers may be preventable

It has been known for nearly 250 yr that at least some cancers are caused by environmental, occupational, or behavioral exposures, scrotal cancer in chimney sweeps being a famous early example (Pott 1775; excerpted in National Cancer Institute 1963). However, what fraction of all cancers is caused by such exposures and what fraction is caused solely by intrinsic biological processes? Epidemiologists provided a first answer half a century ago using statistical methods and data from cancer registries (Kolonel et al. 1980; Ziegler et al. 1993). They argued that if cancer rates are invariant over time and place, the causes of cancer are probably intrinsic; if rates vary, the causes are likely to be extrinsic. Because cancer incidence increases dramatically with age, to compare incidence (or death) rates for specific types of cancers in different countries or within a single country over time, epidemiologists calculate “age-adjusted” rates for a “standard population” with a constant age distribution.

Using this approach, epidemiologists made the following three key observations: (1) The incidence of different types of cancers varies between countries, often by a factor of ≥10. (2) Genetic differences between populations cannot explain most variation because when people move from one country to another, they acquire the cancer incidences of their adopted country within a generation (or sooner, depending on their age when they move). (3) The incidence of some types of cancer has varied dramatically over time within a single country. Data supporting these observations derive from a number of studies. For example, it has long been known that within a generation, the rates of breast cancer and stomach cancer among Japanese migrants to Hawaii significantly shifts toward the rates in native Hawaiians, away from the rates found in genetically similar individuals remaining in Japan, with changes discernible even in the first generation. The multiethnic cohort (MEC) study has systematically integrated data on ethnicity, genetics, lifestyle, and environment to determine the basis for such changes (Kolonel et al. 2004). A stunning example of variation within a single country is stomach and lung cancers in the U.S. A century ago, stomach cancer was the most frequently diagnosed cancer and the leading cause of U.S. cancer deaths (Crew and Neugut 2006), while lung cancer was extremely rare. By 1950 the two were equally common, as the incidence of lung cancer rose, while that of stomach cancer declined (without deliberate intervention) (Cairns 1978). Today, the incidence of lung cancer is roughly eight times that of stomach cancer in the U.S. Such findings led epidemiologists to the stunning conclusion that “cancer incidence is, to a large extent, determined by environment, and so most cancers should, in principle, be preventable.” (Cairns 1978). It remained to identify the carcinogens and, to the extent possible, remove them.

#### A small number of carcinogens cause a surprisingly large fraction of U.S. cancers and cancer deaths

Identifying human carcinogens can be challenging for many reasons. Exposure to a carcinogen can precede the development of cancer by decades or even a generation and may be influential only at a specific developmental stage (Herbst et al. 1971; Bianchi et al. 1997; Green et al. 2011a; Thun et al. 2012). Although research long focused on mutagens as the cause of cancer, as discussed below, many carcinogens promote cancer through nonmutagenic mechanisms for which assays may not be as readily available. While many carcinogens are mutagens, some of these mutagens are rapidly detoxified in the body; conversely, some nonmutagenic substances are converted to mutagens. Adding to the complexity of assessment, some substances that are noncarcinogenic or weak carcinogens can be potent carcinogens if applied in combination.

Today, the International Agency for Research on Cancer (IARC) of the World Health Organization lists >100 “proven” human carcinogens (IARC Monographs on the Evaluation of Carcinogenic Risks to Humans Volume 100A–F, http://monographs.iarc.fr/ENG/Monographs/PDFs/index.php; Pearce et al. 2015; Guyton et al. 2018). There are two “fortuitous” aspects to the list: (1) In principle, exposure to many of these carcinogens could be reduced or eliminated. (2) A very small number are “supercarcinogens” because they cause a large fraction of U.S. cancer cases and cancer deaths, with several causing particularly incurable cancers. “Supercarcinogens” include smoking, sunlight, several infectious agents, and obesity. Introduction of some of these into the population has led to “cancer epidemics” that last a century or more. These agents are effective because so many people are exposed, exposure is frequent or prolonged, and many can drive more than one step of carcinogenesis. Furthermore, some interact with cocarcinogens to become considerably more potent. We turn now to the major human carcinogens in the U.S. population.

### Major U.S. carcinogens: identification, mechanisms of carcinogenesis, and successes and failures in reducing exposure to them

#### Smoking

##### Identification

**Figure 1.**
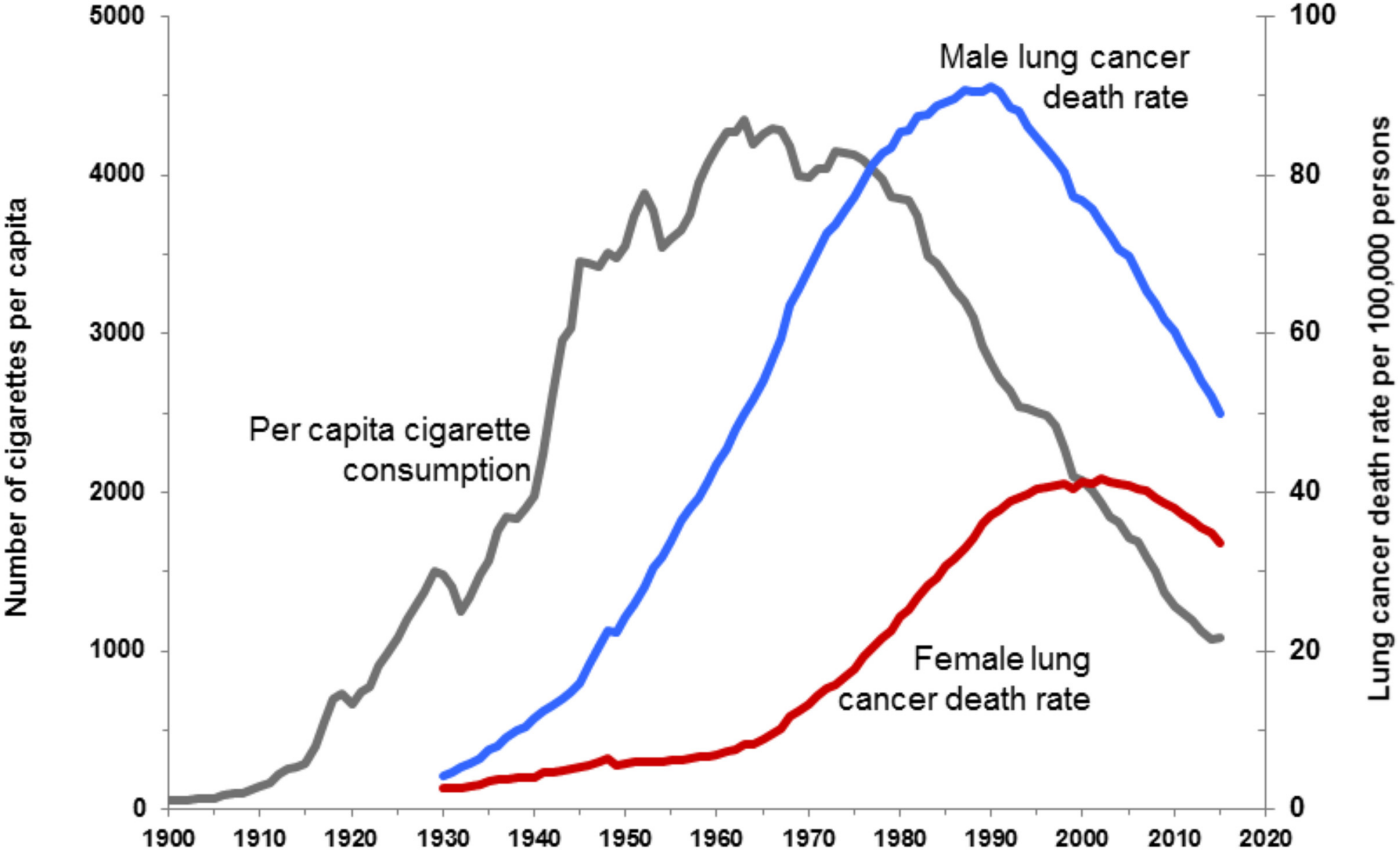
Trends in tobacco use and lung cancer death rates in the U.S. Per capita cigarette consumption versus lung cancer death rates for men and women in the U.S. The figure is reproduced with permission from Cancer Risk Factors and Screening 2018 (https://www.cancer.org/research/cancer-facts-statistics.html), a presentation from the American Cancer Society (American Cancer Society. Cancer Prevention and Early Detection Facts and Figures 2018. Atlanta: American Cancer Society, Inc.). Note that rates are age-adjusted to the 2000 U.S. standard population. Data for death rates are from U.S. Mortality volumes 1930–1959, Mortality Data 1960–2015, National Center for Health Statistics, and Centers for Disease Control and Prevention (https://www.cdc.gov/nchs/products/vsus.htm). Data for cigarette consumption 1900–1999 are from U.S. Department of Agriculture 2000–2015 (Wang et al. 2016).

Before 1900, it was rare for a doctor to encounter cases of lung cancer, but mass production of cigarettes at the end of the 19th Century and the subsequent population-wide merchandising of cigarettes through the mid-20th Century dramatically altered incidence. Smoking was soon suspected as a possible cause of the increase in lung cancer in the early 20th Century, but it was not until 1954 that scientists considered it proven (Proctor 2012). Small case control studies by epidemiologists first showed that lung cancer patients were far more likely to be smokers than noncancer controls. Next, prospective “cohort” studies of two initially healthy groups (smokers vs. nonsmokers), controlling for age, sex, and occupation, showed that smoking 35 cigarettes per day increased the chance of dying from lung cancer by a factor of 40 (Doll and Hill 1954). Meanwhile and subsequently, animal studies showed that cigarette smoke condensates/tars caused cancer when painted on the skin of shaved rabbits or mice, cellular pathologies were detected in the cells of smokers, and, finally, cancer-causing chemicals were discovered in cigarette smoke (U.S. Department of Health and Human Services, https://www.surgeongeneral.gov/library/reports/50-years-of-progress/full-report.pdf). However, there was a significant interval between these conclusions and their wide acceptance by the public, in part due to vigorous advertising campaigns by the tobacco industry and in part because the long 20- to 25-yr lag between smoking uptake and the appearance of lung cancer obscured the relationship (Fig. 1). In the U.S. today, despite a decline in adult smoking rates from 42% in 1965 to 15% in 2015 (Drope et al. 2018), 80%–90% of lung cancer cases and deaths are still due to smoking. Smoking also increases the incidence of cancer in 12–17 other sites, including cancers of the esophagus, larynx/trachea, oral cavity, oropharynx, kidney, bladder, liver, pancreas, stomach, cervix, colon, and rectum and at least one liquid tumor (acute myeloid leukemia [AML]) (U.S. Department of Health and Human Services, https://www.surgeongeneral.gov/library/reports/50-years-of-progress/full-report.pdf). Recent studies estimate that just under 30% of current U.S. cancer deaths are due to cigarette smoking (Alexandrov et al. 2016; Lortet-Tieulent et al. 2016). Although a downward trend in smoking-related cancers continues, due to tobacco control efforts that began in the 1970s and subsequently intensified, there remains much room for improvement.

##### Mechanisms of carcinogenesis

**Figure 2.**
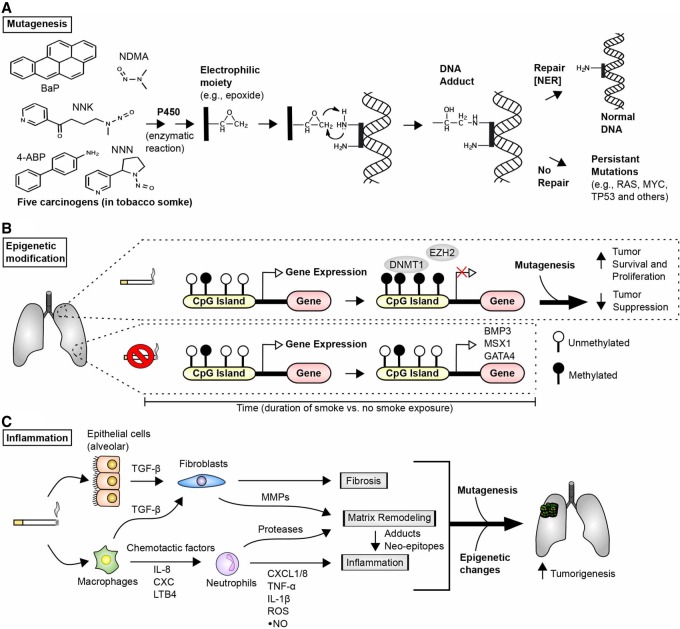
Molecular and cellular responses to tobacco smoke. (*A*) Tobacco smoke contains >70 classified carcinogens (Hecht and Szabo 2014); shown are five compounds strongly associated with mutagenesis: benzo(a)pyrene (BaP), nicotine-derived NNK, N-nitrosodimethylamine (NDMA), 4-aminobiphenyl (4-ABP), and N-nitrosonornicotine (NNN). Many of the compounds in tobacco smoke are metabolized by cytochrome P450, resulting in molecules with highly reactive electrophilic moieties. (Black bar) Representative molecular structures with electrophilic moieties produced from the chemicals metabolized by P450. Electrophilic moieties can readily interact with DNA to form DNA adducts. DNA adducts can be repaired to correct the obstacle and re-establish “normal” DNA; this is frequently achieved by the cell's repair machinery through a process called nucleotide excision repair (NER). However, if repair is unsuccessful and cells do not undergo apoptosis, permanent procancerous mutations may be established. (*B*) Epigenetic modification commonly refers to processes that do not directly alter genetic information encoded by DNA but rather alter availability of genes for transcription; for instance, by addition of reversible methyl or acetyl modifications to DNA or histones. Chronic exposure to tobacco smoke extensively modifies the epigenome of cells in the affected tissue, with characteristic modifications, including hypermethylation of CpG islands (regions with high occurrence of cytosine and guanine separated by only one phosphate group, frequently found near gene promotors). This hypermethylation, generally in the context of tobacco-induced mutations, leads to reduced expression of genes important for tumor suppression and has been shown to significantly contribute to lung tumor formation (Vaz et al. 2017). Methylated residues (filled black circles) are typically generated by the action of methyltransferase enzymes (e.g., DNMT1 and EZH2) and limit transcription of growth inhibitory proteins. (*C*) Tobacco smoke also induces an inflammatory response that involves both epithelial and immune cells. Chemicals in the smoke induce production of fibrosis-associated proteins, most prominently TGF-β (transforming growth factor β); a number of highly active cytokines and regulators of the immune system (e.g., IL-8, C-X-C motif chemokine proteins [CXC], TNF-α, and others); and the release of nitric oxide (NO). This induces fibrosis and remodeling of the extracellular matrix (ECM), creating a more favorable microenvironment for tumorigenesis. (MMPs) Matrix metalloproteinases; (LTB4) leukotriene B4.

The molecular basis for the carcinogenicity of smoke has been analyzed in detail (Hecht 2012; Joehanes et al. 2016), and the following three distinct mechanisms have been identified or implicated: mutagenesis (Fig. 2A), epigenetic modification (Fig. 2B), and inflammation (Fig. 2C). At least 60 known or suspected carcinogens are present in cigarette smoke, including polycyclic aryl hydrocarbons (PAHs) and nitrosamines such as 4-(methylnitrosamino)-1-(3-pyridyl)-1-butanone (NNK). These and other compounds are processed by enzymes of the cytochrome P450 system as part of a detoxification process to generate more water-soluble metabolites that can be secreted. Some of these metabolites are highly reactive due to the addition of electrophilic moieties and form specific types of DNA adducts. Errors during repair of the adducts by the DNA repair machinery generate base changes, with C > A/G > T transversions most strongly associated with smoking-associated lung cancer and responsible for the high mutational burden of these cancers as well as specific hot spot mutations in the *TP53* tumor suppressor gene that are more common in lung cancer than any other form of cancer and useful for supporting a causal relationship between smoking and cancer (Pfeifer et al. 2002). A similar spectrum of mutation is seen in smoking-related cancers arising in a physically proximal location (the larynx), but, in other tissues, different mutational spectra are associated with smoking, suggesting that distinct carcinogens may be more important as mutagens in tissues less directly exposed to smoke.

In support of this idea, a number of recent studies have used genomics to classify mutations in smoking-associated cancers. One study of 5243 cancer genomes (of which 2490 were from individuals known to be tobacco smokers, and 1063 were from individuals who never smoked) identified five mutational signatures elevated in smokers. Of these, the PAH benzopyrene signature was dominant in lung and laryngeal cancer and found to a lesser degree in tissues only indirectly affected by smoking. In bladder, cervical, kidney, and pancreatic cancers, this signature was absent; instead, these cancers were characterized by signatures associated with the APOBEC (apolipoprotein B mRNA-editing catalytic polypeptide) deamination machinery and a “clock-like” signature known to occur over time in many tissues as a correlate of aging (Alexandrov et al. 2016). An independent 2017 analysis of >1000 cancer genomes that used a different algorithm identified three distinct mutational signatures associated with smoking in cancers arising from the kidney and bladder, lung adenocarcinoma, cervical cancers, and squamous cell cancers (lung and HNC) (Supek and Lehner 2017). Complementing this work, studies with mouse models have directly confirmed that treatment with distinct mutagens found in cigarette smoke leads to different mutational signatures in lung cancers associated with mutation of the common driver oncogene *KRAS* (Westcott et al. 2015).

In addition to mutagenic effects, metabolites arising from cigarette smoke induce other pernicious changes that promote tumor formation. These include reprogramming patterns of chromatin and DNA methylation and gene expression (Russo et al. 2005; Beane et al. 2007; Scesnaite et al. 2012; Joehanes et al. 2016) affecting many genes, including some known to have tumor-suppressive function. Smoking also induces a proinflammatory environment that is thought to be able to promote lung cancer. Many smokers develop chronic obstructive pulmonary disorder (COPD), a common comorbidity and cancer-predisposing condition characterized by fibrosis, oxidative stress, and other changes, causing secretion of EGF, IL-1, IL-8, TGF-β (transforming growth factor β), and other inflammatory cytokines, chemokines, and growth factors (Fig. 2C; Young et al. 2009; Adcock et al. 2011). These factors help promote lung cancer pathogenesis, with one of the more intriguing recent clinical results being the observation that the IL-1β inhibitor canakinumab, in a trial assessing activity in reducing atherosclerosis, showed unexpected efficacy in reducing the incidence of lung cancer (Ridker et al. 2017). In contrast to the mutational effects of smoking, epigenetic and inflammatory consequences are reversible, presumably contributing to the reduced risk of lung cancer observed in smokers who quit (Chen et al. 2016).

Although smoking is an independent risk factor for many cancers, it commonly acts with one or more chemical exposures that affect the lung and other tissues. These cocarcinogens include workplace or other exposure to agents such as asbestos, which is associated with common forms of lung cancer as well as a specific risk for mesothelioma. Like smoking, asbestos exposure triggers characteristic genetic (Borczuk et al. 2016) and epigenetic (Nymark et al. 2011; Kettunen et al. 2017) changes that are distinct from those found with smoking but also contribute to malignancy. The interaction of cocarcinogens with tobacco smoke can be complex, making it difficult to quantify individual risk.

##### Successes and failures in eliminating cigarette smoking

**Figure 3.**
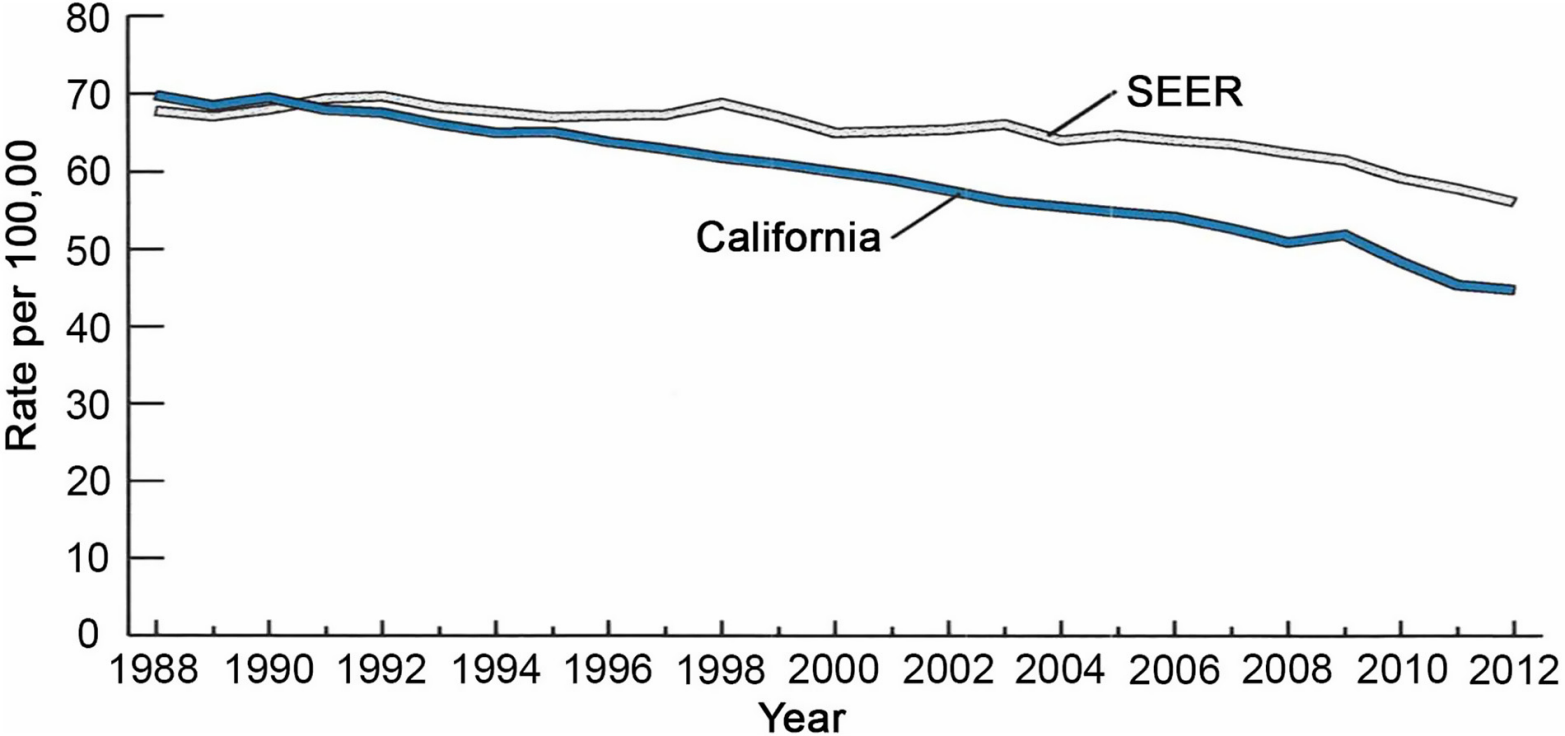
Trends in lung cancer incidence in California and Surveillance, Epidemiology, and End Results (SEER) (SEER) areas other than California, 1988–2012. The age-adjusted incidence of lung cancer in California versus in areas of SEER data other than California. The greater decline in incidence in California correlates with strong anti-smoking policies that have led to lower rates of cigarette smoking in California than in most other parts of the U.S. The figure is taken from California Facts and Figures 2016 (http://www.ccrcal.org/pdf/Reports/ACS_2016_FF.pdf). Note that rates are age-adjusted to the 2000 U.S. population. Data are from the American Cancer Society, California Department of Public Health, and California Cancer Registry. Data for Oakland, California, are from American Cancer Society, Inc., California Division, 2016 (https://www.cdph.ca.gov/Programs/CCDPHP/DCDIC/CDSRB/Pages/California-Cancer-Registry.aspx).

The reduction in U.S. smoking rates is a major accomplishment and a major contributor to declining U.S. cancer death rates, although, as noted above, smoking still accounts for nearly 30% of cancer deaths in the U.S. (Alexandrov et al. 2016; Lortet-Tieulent et al. 2016). Success depends on a comprehensive tobacco control program that includes a combination of evidence-based policies (e.g., raising tobacco taxes and smoke-free air policies), public educational campaigns, and programs to prevent smoking uptake and aid cessation (The Community Guide, https://www.thecommunityguide.org/content/comprehensive-tobacco-control-programs-reduce-tobacco-use; the American Cancer Society, https://tobaccoatlas.org/wp-content/uploads/2018/03/TobaccoAtlas_6thEdition_LoRes_Rev0318.pdf; U.S. Preventive Services Task Force 2017, https://www.uspreventiveservicestaskforce.org/Page/Document/RecommendationStatementFinal/tobacco-use-in-adults-and-pregnant-women-counseling-and-interventions1). Nevertheless, the problem is far from solved, and progress is uneven. Smoking rates are inversely correlated with years of education and socioeconomic status (SES) and vary significantly by state (Jamal et al. 2016, 2018). Utah, Colorado, Montana, and California have some of the lowest rates of smoking and cancer mortality; Mississippi, Oklahoma, and Kentucky have some of the highest (Islami et al. 2015). The former is due in part to the cost of combatting marketing campaigns by tobacco companies that target vulnerable populations, while the latter is due to differences in policies among states, particularly taxation to raise the cost of cigarettes. California has been particularly proactive in its comprehensive tobacco control program, resulting in a lower smoking prevalence, which correlates with an incidence of lung cancer lower than the national average (Fig. 3). If current efforts to prevent uptake or support cessation were abandoned, smoking rates could quickly rebound. In addition to sustained application of proven methods, new measures are much needed, such as reducing the allowed limit of nicotine in cigarettes below an addicting dose and continued research on drugs to help combat nicotine addiction. Although the U.S. has had success in reducing smoking rates over the last 50 yr, rates remain high in certain states and population subsets, and, globally, smoking is not yet well controlled; for example, rising smoking rates in Asia are projected to kill perhaps a billion people in the 21st Century.

#### Viruses and bacteria

When the war on cancer began, many suspected that viruses would prove to be a major cause of human cancers. Retroviruses were considered the most likely to be involved, given their long-known role in cancers of mice and chickens (Gross 1953; Weiss and Vogt 2011). However, intense efforts turned up only one such human retrovirus, human T-lymphotropic virus (HTLV), the cause of a rare T-cell leukemia (Gallo 2005), and viruses temporarily lost status as a major cancer risk factor. Today, most people are surprised to learn that persistent virus infections are estimated to cause 20% of cancers worldwide, with the fraction varying from a few percent in the U.S. to 80% in some African countries in which AIDS acts as a cofactor (Plummer et al. 2016). In the U.S., the major cancer-causing viruses are human papillomaviruses (HPVs), which cause cancers of the cervix, oropharynx, and several other sites (e.g., Walker et al. 2017), and hepatitis B virus (HBV) and hepatitis C virus (HCV), which cause liver cancer. Although fewer bacteria have been linked to cancer, *Helicobacter pylori* was identified as a potential causative agent >30 yr ago (Marshall and Warren 1984) and shown to increase risk of gastric cancer in 1991 (Parsonnet et al. 1991); it remains a major contributor to global rates of stomach cancer.

##### HPVs: identification and mechanisms of carcinogenesis and elimination

The initial recognition that HPV infection was associated with early stages of cervical cancer formation was made in 1976 (Meisels and Fortin 1976; zur Hausen 1976). Subsequently, a similar association of HPV infection with a subset of HNCs was made in 1983 (Syrjanen et al. 1983), followed by determination of a causal role of HPVs (Gillison et al. 2000) and the recognition that HPV-associated HNCs had a distinct disease prognosis versus other forms of HNC typically caused by the use of tobacco and alcohol (Gillison et al. 2008). Early work (for review, see Lowy et al. 1994) established that there are a great number of distinct HPV strains, with tropism to distinct tissues, including the skin and the genital and/or oral mucosa. Some of these strains are low risk and are associated with nonlethal outcomes, including genital warts, polyps, and benign lesions of the oral cavity. However, persistent infection with a subset of strains poses high risk for cervical, anogenital, and oropharyngeal cancers and is associated with ∼5% of total cancers (Schiller and Lowy 2012). Among the high-risk strains, HPV16 and HPV18 were the first recognized in studies of cervical cancers (Van Den Brule et al. 1991; Burger et al. 1996); some other strains (e.g., HPV31 and HPV45) are also associated with high risk. The relationship of the virus to cancer is complex, as there are many more individuals infected with HPV than develop cancer. Based on a large 2005–2006 study of nearly 5000 women, ∼40% were seropositive for any one of nine HPV strains evaluated, with 21% seropositive for the high-risk HPV16 and HPV18 strains (Liu et al. 2016).

**Figure 4.**
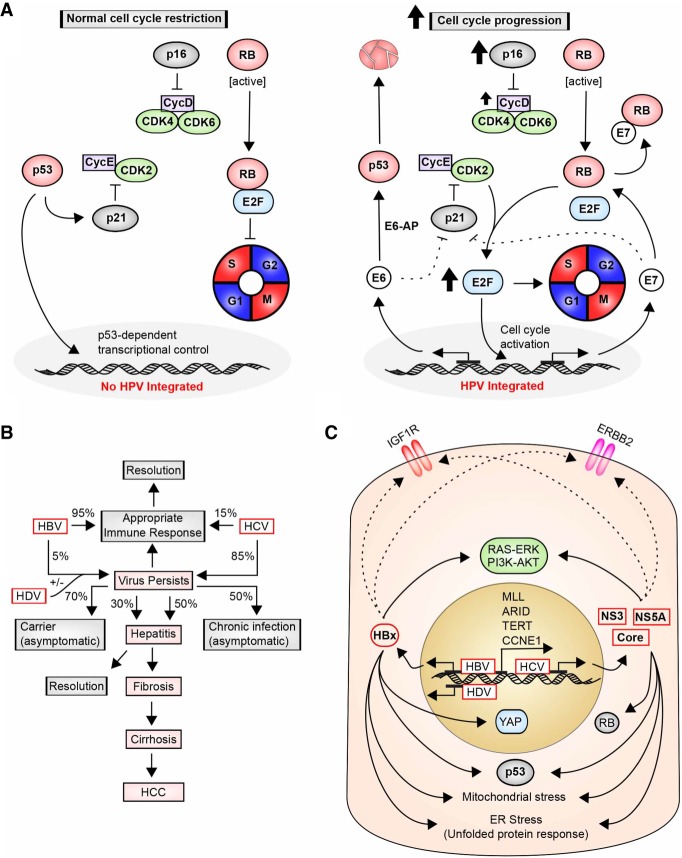
HPV, HBV, and HCV induction of cancers; molecular pathways. (*A*, *left*) In normally growing cells, the action of two tumor suppressors, p53 and RB, is critical in timing the cell cycle and limiting cell growth. p53 activity involves induction of growth inhibitory proteins such as p21/CDKN1A, which inhibits CDK2/Cyclin E and direct DNA binding and transcription; RB sequesters the essential S-phase-promoting transcription factor E2F. Timed inhibition of p53 and RB activity allows progression beyond G1. (*Right*) During chronic infection, HPV integrates its genomic material into the genomic material of human cells, allowing production of viral proteins. Two HPV-associated proteins critical for cell transformation are E6 and E7, which disrupt the core machinery that regulates cell cycle progression. E6 binds to E6–AP to promote the degradation of the tumor suppressor p53. E7 directly targets and disrupts the function of RB and related proteins, causing the release of E2F transcription factors, which promote transition to S phase and thereby drive cell proliferation. Some reports also suggest that E6 and E7 disrupt the inhibitory activity of p21 on CycE–CDK2 in a p53-independent manner. Notably, low RB levels correlate with an increase in the p16/CDKN2A growth inhibitory protein, commonly used as a biomarker to diagnose HPV-positive cancers; however, this does not result in cell cycle block because of additional genomic changes in HPV-positive cancers, such as elevated expression of Cyclin D. (*B*) Infection with HBV and HCV is a common cause of hepatocellular carcinoma (HCC). The percentage of cases in which the virus is not cleared adequately by the immune system varies greatly between HBV (5%–10%) and HCV (85%), with a subset of chronic cases progressing to hepatitis, cirrhosis, and eventual development of HCC. Hepatitis D virus (HDV) requires HBV coinfection but increases the risk of cancer in cases where the viruses are coincident. (*C*) During carcinogenesis, HBV regulatory X protein (HBx) and the HCV NS3, NS5A, and other core proteins activate prosurvival proproliferation receptors (insulin-like growth factor 1 receptor [IGF1R] and ERBB2) and downstream signaling pathways (RAS–ERK and PI3K–AKT). Furthermore, infected cells experience elevated levels of mitochondrial stress and endoplasmic reticulum (ER) stress (Wallace 2012; Clarke et al. 2014). Integration of HBV also induces transcription of important regulatory genes, including MLL, ARID genes, TERT (telomerase reverse transcriptase), and CCNE1 (CycE). HBV and HCV infection has also been shown to repress p53 expression and, in the case of HCV, RB. YAP, an important oncogene frequently elevated in HCC, has been linked to HBx.

HPVs propagate as circular DNA viruses that exist as either chromosome-associated episomes or sequences integrated into the chromosomes of chronically infected hosts. The 8-kb HPV genome encodes nine genes. Only high-risk strains of HPV transform keratinocytes (Schlegel et al. 1988), suggesting unique oncogenic features (Fig. 4A). These features depend on expression of the specific molecular variants of the E6 and E7 proteins present in the high-risk HPV strains (Hawley-Nelson et al. 1989). The primary activity of the E6 oncoprotein is its ability to recruit E6AP/UBE3A, a ubiquitin ligase, and subsequently target E6–AP to promote degradation of the *TP53*-encoded tumor suppressor protein p53 (Scheffner et al. 1993) as well as a number of additional growth-suppressive proteins, such as a transcriptional repressor of telomerase (Gewin et al. 2004). This is complemented by the primary activity of E7 in inhibiting activity of the tumor suppressor RB1 (Munger et al. 1989) and other RB family proteins (Pang et al. 2014). E7 disruption of the DREAM (DP, RB-like, E2F, and MuvB) complex, which represses the expression of cell cycle inhibitors, including *CDKN2A*/p16, accompanied by release of the E2F transcription factor, causes cells to continuously cycle (Johnson et al. 1993; Sadasivam and DeCaprio 2013). Genomic analyses indicate specific features of HPV-associated tumors, some of which (such as chromosome 13q loss and 20q gain) are common to both cervical and oropharyngeal cancers (Wilting et al. 2009). Oropharyngeal cancers arising from chronic HPV infection have a significantly reduced rate of death and better response to treatment than those arising from mutations induced by tobacco and alcohol use (Ragin and Taioli 2007), which likely reflects differences in additional risk factors and genomic differences characterizing HPV-dependent tumors.

At present, a significant and growing fraction of the ∼12,000 oropharyngeal cancers diagnosed in the U.S. each year is associated with HPV infection (typically HPV16) (Gillison et al. 2015; Siegel et al. 2016). A critical factor influencing the U.S. and global incidence of HPV-associated cancers and the potential for effective deployment of a vaccine is the dependence of the virus on sexual transmission. As discussed below, early detection followed by surgical removal of premalignant lesions has had a dramatic impact on the levels of cervical cancer in the U.S., although, worldwide, cervical cancer remains a major cause of death in women because cytologic screening has not been feasible either economically or practically. However, the fraction of all HNCs in the U.S. that are HPV-positive is increasing, particularly in younger patients. The reasons are thought to include a decline in non-HPV-associated HNCs due to a decline in smoking and changes in sexual practice. From 1984 to 2004, the prevalence of HPV-positive oropharyngeal cancers rose by >225%, with a projection that these tumors might become more common than cervical cancers in the U.S. (Chaturvedi et al. 2011).

Vaccines are available now that could prevent the majority of HPV-caused cancers in the future (see below). In Australia, an aggressive program to immunize first girls (beginning in 2007) and now boys could eliminate these cancers over the next several decades (Ali et al. 2013). In the U.S., uptake of the vaccine lagged in part due to inconsistent and unconvincing provider recommendations and the belief that such protection would encourage promiscuity (Rutten et al. 2017), resulting in vaccination rates of 25%–30% of 13 yr olds completing the HPV vaccine series as of 2015 (Vollrath et al. 2017), with only 43% of U.S. teenagers up to date on HPV vaccinations in 2016 (Walker et al. 2017).

##### Hepatitis viruses: identification and mechanisms of carcinogenesis and elimination

Uncontrolled infection with hepatitis viruses, including HBV, HCV, and hepatitis δ or D virus (HDV), is a primary risk factor for hepatocellular carcinoma (HCC) (Fig. 4B; for review, see McGlynn and London 2011; Shlomai et al. 2014). These viruses pose a growing risk in some areas of the U.S., in part due to the growing population of immigrants from east and southeast Asia, where HBV infections are endemic.

The mechanisms by which these viruses induce HCC differ (Fig. 4B,C). The hepadnavirus HBV (discussed at length in Seeger and Mason 2000) is a small DNA virus that is directly transmitted by blood or sexual contact. Infection of infants or young children with HBV prior to full development of the immune system often leads to chronic infection rather than acute infection followed by viral clearance; in contrast, only ∼5% of individuals infected as adults develop chronic infection. Worldwide, it is estimated that >300 million individuals have chronic hepatitis virus infection (Global Hepatitis Report 2017, http://apps.who.int/iris/bitstream/handle/10665/255016/9789241565455-eng.pdf;jsessionid=D599C6B0363AF0A0CEA55CF221A53995?sequence=1). In chronic infection, the HBV virus is maintained as episomes (known as chronically closed circle DNA [cccDNA]) and/or part or full viral sequences integrated into the genome. Persistent HBV infection in the liver is associated with chronic inflammation, activation of proliferation-associated pathways, and stimulation of angiogenesis as well as alterations in DNA damage response and reprogramming of the epigenome. Some of these changes are attributable to inflammation, as the host immune system continually reacts to the presence of viral antigens. Besides providing an immune stimulus, the virus promotes oncogenesis in two additional ways. First, viral integration into the genome is associated with specific hot spots, including genes encoding the chromatin regulatory proteins MLL, MLL3, and MLL4; ARID1A, ARID1B, and ARID2; cyclin CCNE1; and telomerase reverse transcriptase (TERT) (Fujimoto et al. 2012; Sung et al. 2012) as well as other cell growth regulatory proteins (Fujimoto et al. 2016). The viral promoter induces transcription of genes at the integration locus, promoting changes in chromatin organization and transcription of pro-oncogenic genes. Second, the HBV regulatory X protein (HBx) has been defined as an oncogene (Kim et al. 1991). HBx has a complex cellular function involving transactivation of cellular genes, with attendant changes in epigenetic modification of chromatin and direct modulation of cell survival and stress (including DNA damage) responses. Genes regulated by HBx include the oncogene *YAP*, which is commonly elevated in HCC (Zhang et al. 2012); the tumor suppressor *TP53*, which is inhibited at both the transcriptional and post-translational level (Chan et al. 2016); and other genes that promote tumor growth (Amaddeo et al. 2015).

Although relatively rare in comparison with HBV, the δ virus HDV affects >15 million people worldwide (although, to date, with a limited prevalence in the U.S.) (Hughes et al. 2011; Botelho-Souza et al. 2017). The presence of HDV strongly increases the risk for HCC over infection with HBV alone (Ji et al. 2012). This very small virus has a circular RNA genome of 1.7 kb encoding only a single viral protein, the δ antigen (HDAg), and viral transmission is entirely in the context of coinfection with HBV, as the HDV envelope is composed of proteins “borrowed” from HBV. HDV coinfection or superinfection into individuals with chronic HBV causes dramatic worsening of cirrhosis and inflammation, based in part on HDAg induction of TGFB1 (encoding the secreted factor TGFβ) and the cell cycle-promoting transcription factor *JUN* (Choi et al. 2007).

In contrast to HBV, the flavivirus HCV is a small positive stranded RNA virus, with a genome of <10 kb encoding only 10 proteins (Munakata et al. 2007; Shlomai et al. 2014). Primarily transmitted in the U.S. by means of intravenous drug use, it establishes a chronic infection in as many as 80% of infected adults. This causes a persistent inflammatory state that promotes liver cirrhosis and, ultimately, HCC. The oncogenic mechanism of HCV has been harder to dissect than that of HBV due to the limited number of robust in vitro and in vivo model systems; there is a debate as to whether the cancer-inducing activity of the virus is driven primarily by chronic inflammation or specific activity of virally encoded proteins interacting with hepatocyte signaling. There is some evidence that some of the three structural and seven nonstructural (NS) proteins encoded by the HCV genome interact directly with proteins relevant to carcinogenesis. For example, the viral structural core protein has been shown to induce formation of ROS (Okuda et al. 2002), the NS5B protein has been shown to bind and promote destruction of the tumor suppressor RB1 (Munakata et al. 2007), and the NS5A protein has been shown to interact with and regulate function of the tumor suppressor proteins p53 and CDKN1A/p21/Waf (Majumder et al. 2001). Other connections have been identified between viral proteins and control of CTNNB1/β-catenin, the DNA repair signaling protein ATM, and the TGFβ receptor (for review, see Shlomai et al. 2014). Together, these changes increase proliferation and impair apoptosis and DNA repair capacity. However, the co-opting of the innate and immune systems is clearly a critical driver of HCV carcinogenesis, with the viral RNA and proteins triggering activation of signaling systems, including the Toll-like receptors (TLRs) (Wang et al. 2009), the *DDX58*-encoded RIG-1 helicase (Saito et al. 2008), and others, and causing the release of a broad spectrum of cytokines and chemokines (for review, see Martinez-Esparza et al. 2015). Cumulatively, these actions promote hepatic inflammation and fibrosis while disabling the ability of hosts to recognize and clear the virus (for review, see Horner and Gale 2013).

For each of the hepatitis viruses, cirrhosis and cancer risk are increased by common and potent cofactors. In Asia and Africa, one of the most studied cocarcinogens has been aflatoxin, a toxin produced by *Aspergillus* fungi growing on nuts and maize (Kensler et al. 2011; McGlynn and London 2011). Aflatoxin metabolites are also associated with characteristic G-to-T transversions and a hot spot *TP53* mutation (at Ser249). A large epidemiological study in China showed that HBV infection with concurrent evidence of aflatoxin metabolites in the urine resulted in a 60-fold increase in HCC versus the general population and sevenfold and fourfold increases versus either HBV or aflatoxin alone, respectively (Ross et al. 1992); there is some evidence that HCV also synergizes with aflatoxin to promote HCC.

Given awareness of the risk and better controls on the food supply, aflatoxin is not a major problem in the U.S. In contrast, however, several other tumor-promoting conditions are common in the U.S. and worldwide. These include coinfection with HIV (Meijide et al. 2017); chronic alcohol use, which increases the risk for HCC fivefold (Morgan et al. 2004); fatty liver steatosis; obesity; and diabetes, as discussed further below as independent risk factors for liver and other cancers.

Perhaps the boldest experiment in cancer prevention yet undertaken is vaccination of the Taiwanese and Chinese populations with HBV vaccine, which is expected to largely eliminate death from HBV-caused liver cancers (Chien et al. 2006; Liang et al. 2009). Beginning in 1984, children in Taiwan were vaccinated against HBV, and, as a result, by 2003–2011, the majority of HCC diagnoses in Taiwan was in middle-aged or elderly individuals, with significant reductions in the rate of diagnosis in the vaccinated generation and greater reductions expected as this cohort continues to age. Meanwhile, between 1985 and 1990, a population-based randomized controlled trial in Qidong, China, that involved vaccination of half of ∼72,000 newborns demonstrated a significant decrease in the HBV surface antigen seroprevalence and the risk of primary liver cancer in the vaccinated group. As for the U.S., in considering the population-level cancer risk associated with hepatitis viruses, age, geographic location, and access to health care are potent modifying factors. For example, although recommended as part of a standard infant vaccination regimen (Schillie et al. 2018), HBV vaccines are not universally administered for a variety of factors, including parental objections (Sutnick et al. 1971; Lustbader et al. 1976; Szmuness et al. 1980; Tarocchi et al. 2014; A National Strategy for the Elimination of Hepatitis B and C: Phase Two Report, http://nationalacademies.org/hmd/Reports/2017/national-strategy-for-the-elimination-of-hepatitis-b-and-c.aspx).

Drugs that effectively treat HCV infection, such as Mavyret (glecaprevir/pibrentosir) and Epclusa (sofosbuvir/velpatasvir) exist (Bourliere et al. 2017; Forns et al. 2017) but are not yet in broad use due to insufficient population-wide screening for HCV and costs. Treatments for HDV are only now emerging (Elazar and Glenn 2017). In contrast to vaccines, broader use of such tools for secondary prevention is likely to result in a more rapid reduction in liver cancer incidence and death.

##### H. pylori

Gut colonization with some species of microbe, such as *H. pylori*, is a strong risk factor for gastritis, gastric ulcers, and stomach cancer. *H. pylori* is a Gram-negative bacillus that is often present asymptomatically. Rates of *H. pylori* prevalence vary globally; although lower in the U.S. than in developing countries, it is present at higher rates in some U.S. populations, including individuals that are poor and have African ancestry (Goh et al. 2011). The cancer-promoting activity of *H. pylori* is due to expression of bacterial proteins that induce a pathogenic inflammatory response; of these, the cytotoxin-associated A (*cagA*) gene has been most studied, and *cagA*^+^ strains of *H. pylori* are much more cancer-promoting than *cagA*^−^ strains (Kuipers et al. 1995; Peek and Crabtree 2006), based on roles of cagA in reprogramming cancer-associated processes, including cell cycle, cell motility, epithelial–mesenchymal transition, and others (Backert and Blaser 2016). Adding further nuance, the oncogenic properties of geographically distinct cagA proteins differ. For instance, gastric cancer induced by *H. pylori* is high in East Asian countries, and cagA in East Asian strains of *H. pylori* is more effective than cagA from Western strains in activating the SHP2/PTPN11 phosphatase (Hayashi et al. 2017), which triggers the proproliferative kinase ERK1. Proinflammatory stimuli or polymorphisms that trigger interleukin expression are potent cofactors for the procarcinogenic activity of *H. pylori* (Bockerstett and DiPaolo 2017). Interestingly, some recent work has shown that an *H. pylori* infection itself alters expression of genes associated with inflammatory response and affects the composition of the gut microbiome (discussed further below; Kienesberger et al. 2016), providing additional means by which this pathogen may influence colon cancer risk. The striking decline in stomach cancer incidence in the U.S. is attributed to a decline in *H. pylori* in the population, which is thought to be due to improved sanitation, refrigeration, and food preservation as well as use of antibiotics to effectively clear *H. pylori* infections (Crew and Neugut 2006). Eradication of *H. pylori* infection is a major prevention goal, although antibiotic resistance is a significant issue in some populations (Graham and Dore 2016; Marcus et al. 2016).

#### Lifestyle factors: identification and mechanisms of carcinogenesis and elimination

A group of cancer risk factors that includes obesity, diet, and physical inactivity was first identified in affluent industrialized countries and is seen increasingly in populations around the world. These “lifestyle” causes of risk frequently co-occur in individuals: In the past, they have been collectively referred to as the “Western lifestyle” or “affluent lifestyle,” although their increasing prevalence globally and in less affluent populations unfortunately makes such a designation too limiting. Epidemiologists attribute a significant fraction of U.S. cancers to lifestyle factors, and, at current rates, lifestyle factors are expected to surpass smoking as the leading causes of cancer in the U.S. in the first half of this century (Wolin et al. 2011; Colditz and Wei 2012; Song and Giovannucci 2016).

##### Obesity

**Figure 5.**
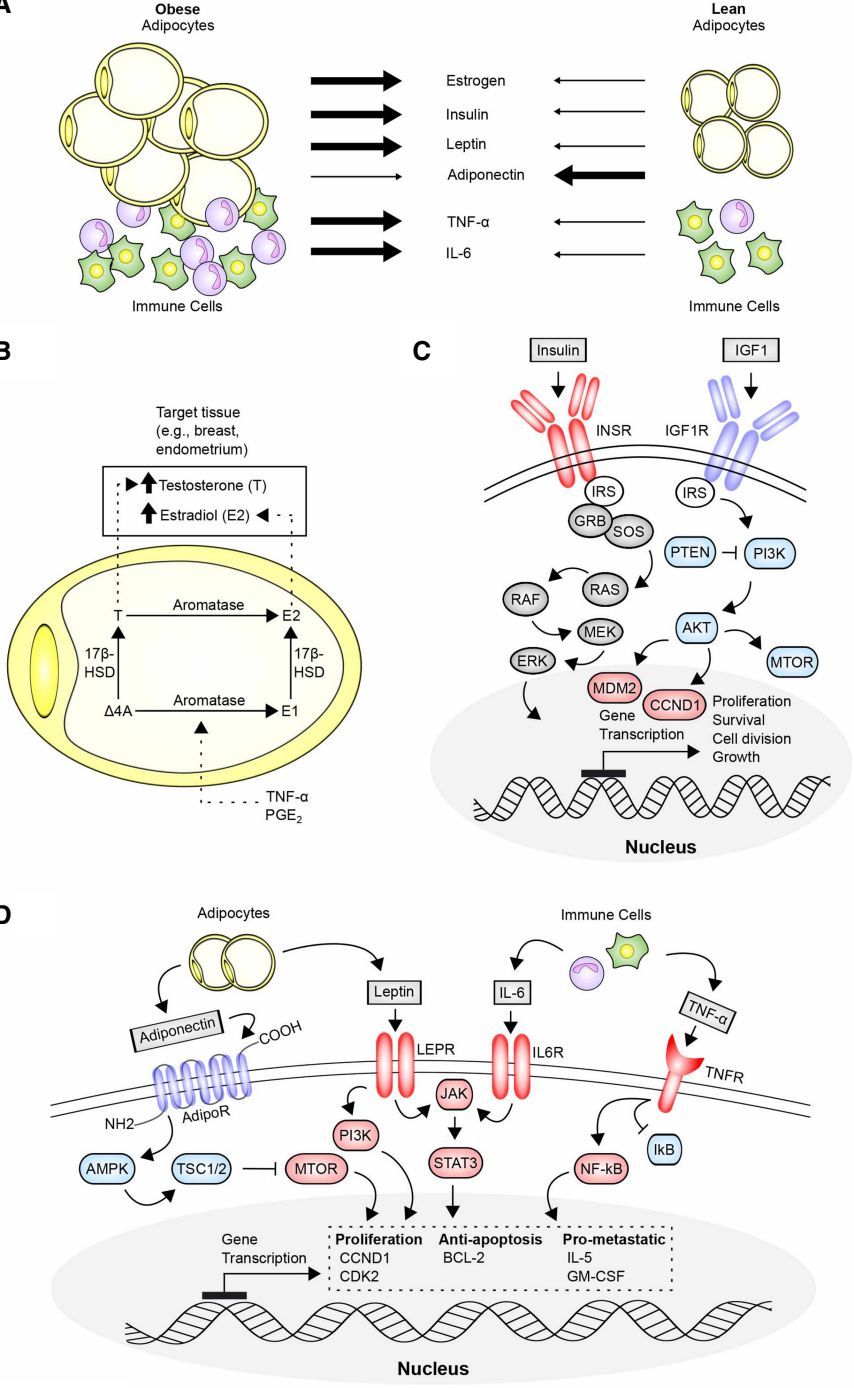
Cancer related molecular mechanisms driven by obesity. (*A*) Obesity causes formation of an expanded compartment of fat-storing cells (adipocytes) and accumulation and activation of immune cells. Signaling from these cells is commonly associated with an increase in TNF-α and IL-6 activity, two important regulators of cell proliferation and inflammation, and is related to immune cell activation. Obesity is also associated with an increase in the level of hormones, such as estrogen, insulin, and leptin, and a decrease in adiponectin, an important negative regulator of metabolism. (*B*) Excess adipocyte activity increases levels of bioavailable testosterone (T) and estradiol (E2) throughout the body. Within adipocytes, androstenedione (Δ-4A) is modified to testosterone by 17β-hydroxysteroid dehydrogenases (17β-HSD) or to estrone (E1) by aromatase (an enzyme also known as estrogen synthetase). In obesity, elevated aromatase expression is facilitated in part by increased levels of prostaglandin (PGE_2_) and TNF-α. In certain tissues (e.g., breast and endometrium), high levels of testosterone and estradiol have been associated with cancer growth and survival. (*C*) Obesity elevates levels of insulin and IGF1, which bind receptors (INSR and IGF1R) expressed on tumor cells to activate core signaling cascades mediated by RAS and PI3K–AKT–MTOR that strongly drive cell proliferation and survival. (*D*) In the obese state, low levels of adiponectin reduce activity of AMP kinase (AMPK), a strong regulator of metabolic activity, particularly relevant in terms of the regulation of MTOR. At low levels of adiponectin, MTOR activity contributes to increased cell proliferation, anti-apoptotic activity, and expression of prometastatic genes. Adipocyte-produced leptin and immune cell-produced IL-6 activate JAK–STAT3 signaling to promote cell proliferation and survival. TNF-α inhibits the negative NF-kB regulator IkB, freeing NF-kB to further support a progrowth state.

Obesity typically arises when the intake of calories exceeds metabolic needs for tissue homeostasis over long periods of time. In 2012, the Annual Report to the Nation on cancer, representing a combined effort of the National Cancer Institute (NCI), the Center for Disease Control (CDC), the American Cancer Society (ACS), and the North American Association of Central Cancer Registries (NAACCR), emphasized the growing impact of obesity (and the frequently concomitant lack of physical activity) on rising cancer incidence for a number of cancer types (Eheman et al. 2012). In 2016, the IARC declared that there is sufficient evidence for a cancer-promoting effect of excess body weight for 13 cancer sites (Lauby-Secretan et al. 2016). Obesity increases the risk and worsens the prognosis for multiple cancer types, including breast cancer, gastrointestinal cancers (pancreatic, colorectal, and esophageal), genitourinary cancers (prostate, endometrial, and renal), and some hematological cancers (multiple myeloma and lymphoma) (Yang et al. 2016). Although not known to be mutagenic or involved in cancer initiation, the obese state triggers changes in cell signaling and metabolism that influence tumorigenesis in several discrete and complementary ways (for detailed review of mechanisms, see Font-Burgada et al. 2016; Hopkins et al. 2016; Iyengar et al. 2016). A critical factor is expansion of white adipose tissue, which stores excess calories as lipids and accumulates in deposits in the breast and viscera with the following effects (Fig. 5):

- Adipose cells produce TNF-α, IL-1β, and prostaglandin-E, stimulating production of aromatase, an intermediate and rate-limiting enzyme in the production of estrogens (Irahara et al. 2006; Subbaramaiah et al. 2012); increased aromatase production by these cells in obese individuals enhances the levels of estrogen available in the breast and endometrium, enhancing cell proliferation and suppressing apoptosis in the breast, endometrium, and other tissues and promoting cancer in older individuals. In the young, the higher estrogen levels associated with obesity induce early age of menarche. Early menarche, like nulliparity or late age of first pregnancy, increases the number of menstrual cycles, which has been associated with increased breast cancer risk (Collaborative Group on Hormonal Factors in Breast Cancer 2012).
- Adipose cells in obese individuals also increase production of the adipokine leptin (associated with regulating appetite), which induces phosphoinositol-3-kinase (PI3K), JAK/STAT, and MEK/ERK signaling, and decrease production of adiponectin, an activator of the AMP kinase (AMPK), which inhibits mTOR and negatively regulates the cell cycle.
- Expanded pools of adipose cells also increase circulating levels of insulin and IGF1. These bind receptors (IR and IGF1-R) on the surface of nonadipose cells, providing another stimulus activating PI3K/AKT survival signaling and concurrently up-regulating *MYC* and other transcription factors that promote cell proliferation. IGF1 and insulin also increase cellular uptake of glucose. This provides additional fuel for cancer cells, which shunt additional glucose to the pentose phosphate pathway (PPP) for catabolism, augmenting production of nucleotides and NADPH and enhancing macromolecular synthesis (a process related to the Warburg effect).
- In obese individuals, adipose cells both directly and indirectly elevate production of a number of chemokines and cytokines. Direct secretion of IL-6 contributes to the activation of proliferative signaling cascade in adjacent tissues. Indirectly, adipose tissue that has become hypertrophied due to obesity creates a proinflammatory environment as regions become hypoxic and die, triggering the release of factors that attract immune cells, which in turn produce additional cytokines. Increased adiposity is associated with production of free fatty acids; these cause stress in the endoplasmic reticulum, promoting the unfolded protein response (UPR) and triggering cell death, separately enhancing the inflammatory microenvironment (Yang et al. 2015).
- Adiposity-associated cytokines have broad action relevant to cancer; for example, experiments in a mouse model suggested that elevated IL-5 and GM-CSF (granulocyte–macrophage colony-stimulating factor) in the lungs of obese mice conditioned the microenvironment, enhancing the homing of mammary tumor metastases to this site (Quail et al. 2017). In parallel, obesity-induced changes in myofibroblasts change the physical architecture of the stroma and extracellular matrix (ECM) surrounding nascent tumors, providing a mechanical cue that supports tumor growth (Seo et al. 2015).
- Obesity and aging have been associated with reprogramming the epigenome via an altered methylation profile; this reprogramming has been associated with changes in gene expression and increased cancer risk, with caloric restriction reversing some of these changes (Coleman 2016; Maegawa et al. 2017).

Taken together, these many actions make obesity a potent accelerant of aggressive tumor growth.

##### Diet

In addition to excess calories, many individuals consume a diet that includes high levels of meat and carbohydrates but fewer fruits, legumes, and vegetables. These characteristics shape the abundance of different species of microbes colonizing the gut and forming the microbiome. In the past decade, appreciation and understanding of the microbiome as an influence on multiple aspects of cancer biology ranging from early tumor formation to response to cancer therapies has greatly expanded. In U.S. populations, a mixture of species of bacteria and fungi predominate in the gut microbiome, although, in at least some populations, protozoa are also present (Chudnovskiy et al. 2016). In contrast to *H. pylori*, some microbes now implicated in contributing to cancer have more subtle actions, modifying the tumor microenvironment without inducing acute gross pathological symptoms, as discussed below.

One important study defined the process of tumor-elicited inflammation (TEI), in which infiltration of gut microbes between epithelial cells lining the colon as tight junctional barriers is disrupted in early adenomas, contributing to establishment of a localized inflammatory response in which initial response to the microbes by the innate immune system recruits myeloid cells that amplify the response, driving tumor progression (Grivennikov et al. 2012). Subsequently, different species of gut microbes were found to have a greater or lesser capacity to induce TEI. The importance of inflammation as a driver of colorectal cancer is now well established and is one reason for the effectiveness of some nonsteroidal anti-inflammatory drugs (NSAIDs) in reducing cancer risk (Todoric et al. 2016). In an exciting recent report, biofilms containing two specific species of bacteria were implicated in specifically elevating inflammation and increasing tumor formation in mouse models and in patients with hereditary risk factors for colon cancer (Dejea et al. 2018).

The contribution of microbes to cancer risk might suggest that extended use of antibiotics might be protective, reducing colon cancer risk by reducing or eliminating gut bacteria; however, a recent study of 16,642 women in the Nurses’ Health Study correlated long-term antibiotic use with increased risk for colorectal adenomas (Cao et al. 2018). The reason for this is not yet understood. Separately, some species abundant in the low-diversity microbiomes associated with diets high in meat and carbohydrates and low in plant-based foods have been associated with increased risk of colorectal cancer (O'Keefe 2016).

In some cases, mouse models were used to show that gut microbiota produce metabolites such as butyrate that can influence DNA repair and histone acetylation and fuel colorectal tumor growth; these activities are favored with specific strains of microbes and a high-carbohydrate diet (Belcheva et al. 2014; Krautkramer et al. 2016). However, interpretation of the activity of metabolites is complex and may vary as a risk factor between different types of cancer and in the context of distinct genetic drivers (for review, see Bultman and Jobin 2014), complicating clear assessment of risk. Lipoteichoic acid (LTA) and deoxycholic acid, obesity-induced metabolites of gut-resident bacteria, have been shown to translocate to the liver and stimulate TLRs, thereby promoting inflammation and HCC progression (Loo et al. 2017). Other metabolites produced by specific species of microbes can modify the activity of therapeutic agents, including PD-1 (PDCD1)/PD-L1 (CD274) targeting immunotherapies, in melanomas and other tumor types (Johnson et al. 2016; Gopalakrishnan et al. 2017; Routy et al. 2017). In one striking recent report, a specific type of microbe from the γ-proteobacteria group was found to exist intratumorally in colon tumors in a mouse model. These γ-proteobacteria expressed a specific form of cytidine deaminase that allowed them to metabolize gemcitabine, causing the tumors to be resistant to this common chemotherapy (Geller et al. 2017). Importantly, gemcitabine is commonly used in the treatment of pancreatic cancer, and a parallel analysis of human pancreatic tumors found that 76% (86 of 113) were positive for γ-proteobacteria, suggesting a potential role in regulation of therapeutic response (Geller et al. 2017).

Red meat and processed meat: In addition to the benefit of a diet largely diverse in plant-derived nutrients in supporting a healthy microbiome, such a diet is also typically associated with reduced consumption of red meat and processed meat. A growing body of evidence indicates that a diet rich in these forms of meat raises the risks of some forms of cancer, with the strongest evidence available for colorectal cancer. The IARC performed an exhaustive meta-analysis of >800 epidemiological studies of cancer risk associated with red or processed meat, with data for individuals from multiple countries and of varying ethnicities. These data suggested that consumption of 100 g of red meat per day or 50 g of processed meat per day led in each case to a 17%–18% increase in risk for colorectal cancer (International Agency for Research on Cancer 2018, http://monographs.iarc.fr/ENG/Monographs/vol114/mono114.pdf). In assigning the mechanistic basis for this elevated risk, several factors are likely to be involved. Red meat is typically consumed following cooking at high temperature or following curing or smoking. These processes result in the production of DNA-damaging carcinogens, including N-nitroso compounds (NOCs) (Lewin et al. 2006), heterocyclic aromatic amines (HAAs) (Gross et al. 1993), and, as with tobacco, PAHs (Cross and Sinha 2004). In addition, meat components such as heme iron have been shown to have genotoxic effects in a number of model systems, based on complex mechanisms that include enhanced production of NOCs and peroxidation of lipids (Bastide et al. 2015). It is likely that for individuals, risk from these carcinogens interacts with intrinsic genetic risk factors for colorectal cancer (discussed below) as well as other dietary components.

Alcohol: Many attempts to identify specific items in the diet that increase cancer risk have failed, but alcohol use is a notable exception. Heavy alcohol use has long been associated with cirrhosis of the liver, which is a strong risk factor for HCC, particularly in combination with infection by hepatitis viruses (Martinez-Esparza et al. 2015). However*,* not until late 2017 did the American Society for Clinical Oncology (ASCO) argue the case for considering alcohol as an active causal agent for multiple forms of cancer (LoConte et al. 2018). The statement, which noted that 3.5% of cancer deaths in the U.S. (and 5.8% of cancer deaths worldwide) are due to alcohol, is based on clear roles for alcohol as a tumor promoter in HCC, breast cancer, and multiple tumors of the aerodigestive tract, including the colon, esophagus, larynx, and oropharynx. These conclusions are made after years of mounting evidence from molecular and epidemiological studies (Meadows and Zhang 2015; Ratna and Mandrekar 2017). Specific mechanisms by which alcohol induces cirrhosis and cancers include, first, the metabolism of ethanol to acetaldehyde as part of the detoxification and excretion process (Garaycoechea et al. 2018). Acetaldehyde is a known carcinogen; individuals bearing inactive alleles of mitochondrial alcohol dehydrogenase (e.g., ALDH2*2) have higher levels of blood acetaldehyde and higher risk of esophageal cancer (Yokoyama et al. 1996). The specific risk of alcohol in individuals of East Asian ancestry is linked to abundance of such inactive alleles in these populations. Large-scale genomic studies have shown that alcohol consumption is associated with patterns of somatic mutation associated with increased error-prone repair dependent on POLH. Bin regions of chromatin are associated with specific chromatin marks (e.g., 3′ gene ends, high transcription levels, and H3K36me3 chromatin), reflecting a direct DNA-damaging activity (Supek and Lehner 2017).

In addition, alcohol metabolites include acetate and a number of ROS that can promote both DNA damage and inflammatory conditions through action on tissues exposed to alcohol and via recruitment and activation of immune cells (Wang et al. 2012). The activity of alcohol on white adipose tissue increases its expression of proinflammatory cytokines and chemokines. Alcohol also causes changes in the lining of the colonic epithelium that reduce barrier function, increasing infiltration of microbes and their products into interstitial spaces and increasing inflammation. Furthermore, there is some evidence that chronic alcohol use significantly alters the composition of the gut microbiome in a manner that promotes liver disease (Yan et al. 2011). Chronic alcohol use is also linked to changes in retinoid metabolism, which may be linked to elevated estrogen production (Seitz et al. 2012); it is notable that estrogen receptor-positive breast cancers have been reported as most strongly induced by alcohol consumption (Suzuki et al. 2008).

Dietary supplements: Another common feature of diets of individuals in the U.S. and other Western countries is the use of vitamins. Although safe at low doses, a significant number of people take high doses of vitamins. This is emerging as an unexpected source of risk; for example, high doses of the vitamins B6 and B12 were shown recently to increase the risk of lung cancer in males (Brasky et al. 2017). Older reports also make clear the unanticipated damaging consequences of taking isolated dietary components. For example, the β-carotene and retinol efficacy trial (CARET) was based on the observation that individuals eating many fruits and vegetables rich in these components had lower rates of lung cancer. The CARET evaluated the effect of daily doses of 30 mg of β-carotene and 25,000 IU of vitamin A. This trial was stopped early because of evidence of no health benefits; in fact, there were 28% more lung cancers and 17% more lung cancer deaths in individuals receiving the supplements (Omenn et al. 1996). Similar results were found in the α-tocopherol β-carotene cancer (ATBC) prevention study, in which participants received α-tocopherol and β-carotene (Albanes et al. 1996). Such studies provide strong cautionary lessons.

Although likely affecting a smaller pool of individuals, another dietary source of cancer risk in Western countries is from toxic or mutagenic metabolites found in dietary supplements and natural herbal remedies. Largely unregulated, these products may contain heterogeneous mixtures of ingredients with undefined chemical components; furthermore, the degree of consumption is difficult to track, confounding assessment of the associated risk. However, there are well-documented associations between the ingredients of some components of dietary supplements and clear mechanisms of carcinogenesis. For example, plants of the genus *Aristolochia* have been a common ingredient in some Chinese herbal remedies and other supplements. However, aristolochic acid, a major component of preparations of *Aristolochia*, is a potent mutagen, inducing A-to-T transversions and a unique genomic signature in urothelial cancer, renal cell carcinoma, and HCC (Hoang et al. 2013, 2016; Poon et al. 2013).

##### Physical inactivity

Diet is linked to obesity, and diet and obesity increase cancer risk. The other major factor contributing to risk of obesity and often co-occurring with poor diet is limited physical activity, which may pose a risk independent of obesity per se. Particularly given the strong emerging links between changes in metabolism, adiposity, and tumor energetics, a reasonable hypothesis would be that exercise might influence all of these properties, reducing cancer risk. Some studies are beginning to support this idea, justifying the idea that tumor risk decreases with physical activity or with genetic backgrounds that are associated with “heritable fitness” (Thompson et al. 2017). A large meta-analysis found a significant inverse relationship between incidence of colon adenomas and degree of physical activity (Wolin et al. 2011). A recent comprehensive review (Koelwyn et al. 2017) describes the profound effects of exercise on metabolism, aerobic capacity, angiogenesis, and immune response, resulting in a reprogramming of the tumor microenvironment and strongly justifying further investigations in this area.

Except for excessive alcohol use, there are few if any proven methods for changing the habits that together comprise the lifestyle profile summarized above. Presumably, as with smoking, the most effective approach would be to make the desired behavior the only or the easiest choice; for example, by banning or heavily taxing foods such as large sugary drinks (https://www.theguardian.com/society/2017/feb/2022/mexico-sugar-tax-lower-consumption-second-year-running) and designing cities and workplaces that make physical activity unavoidable. As with smoking, success will likely require comprehensive strategies incorporating diverse approaches, including education, public policy, and research to identify successful ways to modify behaviors at the population level over the long term.

#### Sun exposure: identification, mechanisms of carcinogenesis, and reducing exposure

Approximately 180,000 melanomas will be diagnosed in the U.S. in 2018, as will >1 million cases of squamous cell carcinoma (SCC) and >4 million cases of basal cell carcinoma (BCC) (https://www.cancer.org/content/dam/cancer-org/research/cancer-facts-and-statistics/annual-cancer-facts-and-figures/2018/cancer-facts-and-figures-2018.pdf; http://www.cancer.org/cancer/skincancer-basalandsquamouscell/detailedguide/skin-cancer-basal-and-squamous-cell-what-is-basal-and-squamous-cell). Exposure to the ultraviolet (UV) irradiation from sunlight or artificial sources is the major source of risk for melanoma, especially during childhood and adolescence (Wu et al. 2014), as well as for SCCs and BCCs, in which cancer risks appear to be more closely associated with cumulative UV exposures across the life span (Zaidi et al. 2012). Both UVA (320–400 nM) and UVB (90–320 nM) irradiation contribute to cancer etiology but through nonequivalent means. UVB directly targets DNA, forming cyclobutane pyrimidine dimers (CPDs) between cytosine and thymine residues, leading to characteristic C > T and CC > TT transitions. This signature differs from that induced by other forms of irradiation, such as therapeutically administered ionizing radiation (IR), and from some mutagens (Davidson et al. 2017). Typically, CPD lesions are repaired by nucleotide excision repair (NER) (Sarasin 1999); because NER is blocked by active transcriptional complexes, in melanoma, mutations are enhanced in active promoter regions characterized by DNase I-hypersensitive sites (DHSs) (Perera et al. 2016; Sabarinathan et al. 2016), leading to potential effects on gene expression. Ultradeep sequencing of sun-exposed noncancerous skin from 234 biopsies of eyelid epidermis found two to six mutations per megabase per cell and specific signs of this C > T, CC > TT UVB mutational signature as well as evidence for C > A transversions (Martincorena et al. 2015). Stunningly, this analysis found a “patchwork” effect of clonal variation across 74 analyzed genes, with cancer-associated driver mutations under selection in a heterogeneous manner in 18%–32% of “normal” skin cells (Martincorena et al. 2015). This pattern explains the high incidence, high clonal diversity, and malignancy of melanomas compared with many other tumor types.

A second important means by which UVB promotes malignancy is through engagement of the innate immune system, which depends on activation of TLR3 (Bernard et al. 2012) and TLR4 (Bald et al. 2014), interferon signaling (Zaidi et al. 2011), and other factors (for review, see Zaidi et al. 2012). This proinflammatory environment induces epigenetic changes within the tumor cells and contributes to a tumor microenvironment supporting progression, including angiogenesis and metastasis.

In contrast, the role of UVA has been more difficult to elucidate, although UVA is clearly less potent as a mutagen than UVB. Some data suggest that it acts more indirectly in melanoma induction, generating ROS (Noonan et al. 2012). This study established that UVA increased levels of 8-oxo-7,8-dihydro-2′-deoxyguanosine (8-oxodGuo), a mutagenic metabolite that can cause GC > TA transversions characteristic of ROS, in UVA irradiated melanocytes. Curiously, induction of this species is dependent on the presence of melanin, which was proposed to serve as a source of melanin radicals, providing an oxidant function. Additional UVA activities are likely; significant work is required to unravel the relevant mechanisms (Karran and Brem 2016).

A success in cancer prevention is the reduction in sun exposure and resulting decline in skin cancer in Australia due to an advertising campaign introduced in 1981 and called Slip Slop Slap that aims to make sensible sun exposure the social norm (Green et al. 2011b). Confounding similar efforts in the U.S. is the rising use of tanning salons, particularly for the young, that has been accompanied by a predictable rise in rates of melanoma and other skin cancers (Boniol et al. 2012; Wehner et al. 2014; The Surgeon General's Call to Action to Prevent Skin Cancer, https://www.surgeongeneral.gov/library/calls/prevent-skin-cancer/call-to-action-prevent-skin-cancer.pdf).

### Additional causes of cancer in the U.S. whose impact is smaller or of unknown magnitude

#### Environmental, occupational, and industrial pollutants

A number of human carcinogens were first identified as a result of the industrial revolution when workers were exposed to high levels of novel chemicals or sources of radiation, resulting in noticeably higher concentrations of cancers (Siemiatycki et al. 2004). These examples, combined with the fact that, in the U.S., many chemicals have been released into the environment without first being tested for carcinogenicity (Judson et al. 2009; Egeghy et al. 2012), led to the serious concern that chronic exposure to industrial pollutants may cause a large fraction of human cancers at the population level. While there is no doubt that they cause some fraction, it is still difficult to know how much. Epidemiological data suggest that it is less than one might suspect, since there was no sharp rise in overall cancer rates that followed the industrial revolution. However, if many pollutants each contributed to a small increase in cancer risk, it would be very difficult to detect by epidemiological analysis alone. The reason that no striking increase in cancer rates was detected may well be due to strong environmental and workplace protections that limit the exposure to industrial carcinogens in the general population as well as for industrial workers. Industrial use of some cancer-inducing agents with clear causation mechanisms, such as asbestos, is now strictly controlled. However, residual contaminated hot spots remain; there are 3000 cases per year in the U.S. of the asbestos-associated cancer mesothelioma, and, globally, carcinogens (including asbestos, arsenic, radon, and other agents) are abundant (Hubaux et al. 2012). Together, clean air, water, and workplace regulations coupled with active surveillance for validated carcinogens help to reduce exposure to these and other highly validated agents.

On the other hand, there are many chemicals used in industrial production, agriculture, and household items that have unknown consequences for, or an unquantified impact on, risk. It is likely that at least some cancers arise from a number of these with thus potentially modifiable exposures. It is nevertheless challenging to definitively assign risk to environmental factors because typically doses of suspected agents are low, and cancers can arise years after the initial exposure. In addition, carcinogens act in different ways, with some providing initiating stimuli (inducing mutation), others primarily acting as tumor promoters (particularly in susceptible individuals) (discussed below), and some doing both.

Current approaches to identifying additional sources of risk build on a large body of past work that has systematically analyzed evidence for the risk of specific carcinogens causing distinct types of human cancer (Cogliano et al. 2011). One effort is profiling old and new carcinogens based on 10 essential features, including the following attributes of an agent: (1) acts as an electrophile either directly or after metabolic activation, (2) is genotoxic, (3) alters DNA repair or causes genomic instability, (4) induces epigenetic alterations, (5) induces oxidative stress, (6) induces chronic inflammation, (7) is immunosuppressive, (8) modulates receptor-mediated effects, (9) causes immortalization, and (10) alters cell proliferation, cell death, or nutrient supply (Smith et al. 2016). These efforts are supported by next-generation sequencing (NGS) and other profiling approaches that allow assignment at a molecular level of profiles associated with risk. These approaches also incorporate data generated by genomic, transcriptomic, and proteomic analysis (Cote et al. 2016); allow comparison with functional testing in animal models (Rudel et al. 2014); and can be benchmarked to characteristic profiles generated for specific mutagens (Alexandrov and Stratton 2014; Nik-Zainal et al. 2015).

#### Medical care

There has been tremendous progress in medical treatment of many chronic and acute diseases. In some cases, although treatments may ameliorate symptoms or actually be lifesaving in the short term, they can also increase the risk of some forms of cancer over the long term. Among the better studied examples, ionizing irradiation used as treatments for cancer increases the risk of secondary cancers of a number of distinct types, characterized by specific genomic signatures (Behjati et al. 2016; Davidson et al. 2017). Given the life-threatening nature of many cancers, the risk imposed by irradiation and many forms of chemotherapy cannot be avoided. However, other forms of medication are elective and are associated with the potentially avoidable risk of either cancer or death due to cancer, although the quantification of this risk is challenging. The use of alternative medicines for cancer treatment falls in this category (Johnson et al. 2018). Another source of risk may arise from the use of hormonal therapies, such as estrogen replacement therapy after menopause, where some cancers, such as breast and ovarian, are elevated with specific formulations of hormones, while risk of other life-threatening conditions, such as heart disease, is reduced (Lobo 2017). In some cases, medications may interact; for example, several intriguing studies have shown that androgen regulates the DNA damage response, influencing response to IR (Polkinghorn et al. 2013; Spratt et al. 2015). These findings raise the possibility that long-term use of androgen deprivation therapy (ADT) or the change in estrogen–androgen balance associated with obesity may potentially alter the risk of cancer-inducing mutations arising from exposure to mutagenic stimuli. Finally, given the long latency of cancer formation and the growing use of new potent medications targeting many distinct classes of cellular signaling protein over the past several decades, it is likely that some of these may be tumor-promoting. The dilution of signal across the general population will make it difficult to identify such risks with current information sources.

### Molecular prevention, interception, and immunization against nonviral cancers

A complementary approach to cancer prevention is the use of drugs or vaccines to inhibit, reverse, or delay the onset or progression of cancer, informed from an understanding of the molecular mechanisms underlying initiation and progression of nonviral tumors. Called “chemoprevention” if the drug prevents development of cancer, “interception” if it delays or reverses the progression of an early stage cancer, or “reverse migration” if it uses a treatment for advanced cancer on an early stage tumor, these approaches are similar in concept to the use of statins to prevent heart disease (Blackburn 2011; Albini et al. 2016). Since drugs may need to be taken for long periods of time by healthy individuals, they must have no or minimal side effects. This field of research is still in its infancy, but several Food and Drug Administration (FDA)-approved agents lead to significant reductions in the incidence of specific types of cancer. Notable are drugs that reduce the incidence of estrogen receptor-positive breast cancers by ≥50%. These include the selective estrogen receptor modulators (SERMs) tamoxifen and raloxifene and aromatase inhibitors such as anastrozole, letrozole, and exemestane (Cummings et al. 1999; Powles et al. 2007; Vogel et al. 2009; Cuzick et al. 2011; Litton et al. 2012; Reimers et al. 2015). None is without side effects, however, so use has to be weighed against individual cancer risk and the fact that early detection and improved treatment of early stage estrogen receptor-positive breast cancers has continued to advance.

Another promising potential agent for cancer prevention is the anti-diabetic drug metformin. Metformin activates AMPK, inducing muscles to take up glucose from the blood and thereby ultimately reducing insulin production. The drug has been widely used for diabetics and found safe. Following the recognition that AMPK is a critical target of regulation by the LKB1 tumor suppressor, investigators performed a retrospective review of clinical data that suggested that diabetic patients receiving metformin might have a reduced risk of cancer (Evans et al. 2005). This initial study was supported by a number of others that reached similar conclusions (Decensi et al. 2010); in the interim, the known mechanism of action of metformin was expanded beyond control of AMPK to regulation of mitochondrial and lysosomal function (Rena et al. 2017). Clinical trials are under way to determine the efficacy of this drug in preventing breast, colorectal, pancreatic, and prostate cancers (Amin et al. 2016; Sayyid and Fleshner 2016; Heckman-Stoddard et al. 2017; Jung et al. 2017). A number of studies have investigated the impact of NSAIDs, including aspirin and COX-2 inhibitors, on the incidence, recurrence, and mortality from colon cancer. These agents can have significant preventive effects yet must be considered in the context of established side effects (Gupta and Dubois 2001; Wang and Dubois 2010; Thompson et al. 2016). As of 2018, the U.S. Preventive Services Task Force (https://www.uspreventiveservicestaskforce.org/Page/Name/recommendations) recommends that adults aged 50–59 without elevated risk of bleeding but with elevated risk for cardiovascular disease take daily low-dose aspirin as a primary prevention against colorectal cancer (https://www.uspreventiveservicestaskforce.org/Page/Document/RecommendationStatementFinal/aspirin-to-prevent-cardiovascular-disease-and-cancer).

Vaccination against viral-induced cancers is one of the spectacular successes of the war on cancer and has the potential to further reduce cancer deaths by 15%–20% worldwide. Could nonviral cancers be prevented by vaccination? Dramatic success in immunotherapy for advanced cancers has invigorated the field. To date, target antigens have been largely patient- and tumor-specific. However, it is possible that common antigens for some types of cancers could be found. The decades-ago discovery that cancers often arise from and remain dependent on mutated oncogenes and tumor suppressor genes and that cells present peptides on their surface for screening by the immune system suggested that it might be possible to immunize against peptides bearing common mutations in cancer-associated genes, such as the tumor-promoting oncogene *RAS* or the tumor suppressor TP53 (in this case, the target would be gain-of-function somatic mutations that act in a cancer-promoting manner). That such mutations can be immunogenic in some major histocompatibility (MHC) contexts has been shown (Tran et al. 2016; Marty et al. 2017), but whether immunization against such mutations will ever be possible is not known. However, a vaccine comprised of multiple immunogenic peptides derived from KRAS showed promising activity in a mouse model of lung cancer (Pan et al. 2017), and it will be of great interest to follow the development of such lines of research.

## Genetic predisposition to cancer: identifying high-risk individuals for prevention, screening, and interception

### Inherited risk factors affecting tumor initiation

When estimating cancer risk and developing strategies for prevention and early detection, it is important to consider that within a general population, the risk of individuals for specific types of cancer can vary significantly based on inherited factors. Over the past two decades, the general understanding of factors causing genetic predisposition to cancer has increased significantly. Earlier studies focused on a small number of genes that were discovered through observations in multiple affected family members (“loaded pedigrees”) and settings with cases enriched in specific populations or ancestries (e.g., Ashkenazi Jews). The variants discovered in such studies were often highly penetrant (forms of genetic variation strongly predisposing to cancer) for a limited number of cancer sites. Examples of genes where inactivating or hypomorphic mutations lead to such strong signals include the *BRCA1* and *BRCA2* genes, variants in which predispose strongly to breast and ovarian cancer (Welcsh and King 2001), and the set of mismatch repair genes associated with Lynch syndrome (*MSH2*, *MLH1*, *MSH6*, and *PMS2*) (Jasperson et al. 2010). Another example for colon cancer involves inherited genetic variants in the *APC* gene that lead to familial adenomatous polyposis (FAP), affecting one in 10,000 individuals and conferring ∼95% risk of colorectal cancer by the age of 50 (Jasperson et al. 2010). Rare mutations in the Fanconi anemia (FA) genes are associated with very high risk of AMLs and HNCs and are at particular risk from UV and other forms of irradiation and tobacco smoke (Romick-Rosendale et al. 2013). For the breast and colon cancers, it is estimated that up to ∼5% of total cancers are associated with such penetrant forms of genetic variation. For carriers of these variants, active surveillance starting at an early age, coupled in some cases with management of lifestyle exposures and prophylactic surgery (e.g., mastectomy/oopharectomy for *BRCA* mutation bearers), is routine (Wei et al. 2010; Kanth et al. 2017).

It is now clear that such relatively common highly penetrant damaging genetic variants represent only a small subset of cancer-predisposing inherited variants. For example, studies of Scandinavian twins and other population-based studies have estimated that up to 30% of the risk for colorectal cancer (including “sporadic” or nonfamilial cases) can be attributed to inherited genetic variation but that these variants may be rare and/or of modest penetrance (Lichtenstein et al. 2000).

Indeed, the majority of risk-associated gene variants in an entire population is expected to be of low to intermediate penetrance. Many of these genes are dispersed across a network of proteins associated with control of the DNA damage machinery, directly targeting either proteins involved in DNA repair (Srivas et al. 2013; Nik-Zainal et al. 2014; Arora et al. 2015; Nicolas et al. 2015; Shlien et al. 2015) or proteins regulating the activity of DNA repair proteins. For example, panel testing for a signature of *BRCA* gene deficiency identified such a signature in 22 tumors with *BRCA1/BRCA2* genetic lesions and 47 tumors without such lesions, likely due to defects in BRCA regulatory proteins (Davies et al. 2017). Some predisposing variants are associated with specific types of cancer and sensitivity to specific controllable factors; for instance, a variant in the *BAP1* gene is predisposing to a small group of cancers, including mesothelioma (Murali et al. 2013) and greater cancer risk from exposure to asbestos (Testa et al. 2011) and other environmental carcinogens (Bononi et al. 2017). Many variants of unknown significance (VUSs) occurring in the general population remain to be assigned for function. Ongoing efforts to characterize the import of such VUSs combine exome analysis and functional testing for phenotypic effects on DNA repair (e.g., see Arora et al. 2015). Large databases, such as ClinVar and others, are systematically compiling information found in affected individuals, the general population, and other populations of interest (for example, the healthy elderly), with the goal of generating a resource that can be used to support statistical estimations of gene–risk correlation (Bodian et al. 2014; Erikson et al. 2016; Rancelis et al. 2017). The NGS technological revolution has driven down the costs required to discover the complete set of genetic variants in large cohorts of individuals, allowing large surveys of variation in the exomes of cancer cases and associated population-based controls. Such studies may yield discoveries of rare forms of variation that confer intermediate (and potentially actionable) levels of risk; such forms of variation would have been missed by large-scale surveys of common variation.

Aside from the daunting number of candidate cancer risk genes to be assessed, a number of confounding factors complicate risk prediction based on analysis of genes that function in an autocrine manner to prevent normal cells from undergoing transformation. For example, some known somatic driver mutations in the genes *ARID1A*, *PIK3CA*, *KRAS*, and *PPP2R1A* have been found in the endometriotic lesions of 19 of 24 patients with deep-infiltrating endometriosis even though this form of endometriosis almost never undergoes malignant transformation (Anglesio et al. 2017; Dawson et al. 2018). How tissue microenvironment versus cell-intrinsic factors restrains the transforming effect of these mutations remains to be established.

### Non-cell-autonomous inherited and acquired traits influencing tumor formation

In contrast to the longtime focus on cancer risk arising from inherited gene variants affecting the cell that becomes the tumor, a growing field of research addresses non-tumor-intrinsic inherited and noninherited features that influence the ability of a mutated cell to progress. Broadly speaking, these changes affect the tumor microenvironment—a compartment composed of multiple untransformed cell types, including both immune system and stromal cells, as well as secreted insoluble proteins of the ECM and associated soluble factors. This vast topic cannot be summarized in any depth here (for reviews, see Lu et al. 2012; Gajewski et al. 2013; Faurobert et al. 2015). We focus on two examples.

#### Immune surveillance

A growing body of data indicates an important role for the host immune system in the surveillance and elimination of cancer cells, with the recognition that escape from immune restriction is an essential transition at early stages of tumor growth (for review, see Dunn et al. 2002; Koebel et al. 2007). This role for the immune system accounts for the well-known elevated rates of multiple forms of cancer in immunosuppressed patients, such as solid organ transplant recipients, where 32 distinct malignancies occur at elevated rates (Engels et al. 2011). In an exciting recent study, Marty et al. (2017) explored the hypothesis that one inherited factor regulating the emergence of tumors is individual variation in the ability of the immune system to recognize specific common transformation-associated mutations (for example, the G12V and G12D mutations of *KRAS* or the R175H mutation of *TP53*). By modifying existing algorithms to study the ability of MHC proteins to present peptides to the immune system, they were able to rank >300 common MHC-1 alleles dispersed in the population for their ability to present peptides bearing such common oncogenic driver mutations. They subsequently compared the co-occurrence of MHC-1 HLA-A, HLA-B, and HLA–C alleles with high or low presentation capacity with specific common oncogenic mutations across 1018 likely driver mutations found in a set of 9176 tumors in The Cancer Genome Atlas. This led to the conclusions that some common drivers are uniformly poorly presented by MHC-1 alleles and also that there existed a strong correlation between an MHC-1 profile associated with poor presentation and the likelihood of the mutation being present in a patient's tumor. This offers a new strategy to qualify the risk associated with specific inherited genetic variants or somatic mutations (Marty et al. 2017), which is potentially relevant to other immune system antigen recognition components. However, in assessing genetic contributions, it is also important to keep in mind the fact that immune system contributions can be potently influenced by the behavioral and environmental factors discussed above. As only one example, inflammatory signals associated with obesity alter the landscape of immune cells in a manner that promotes metastasis (Quail et al. 2017). Finally, the immune system is subject to declining or aberrant function in age (e.g., Palmer et al. 2018; Shlush 2018), making the efficiency of immune surveillance inextricably linked to the process of aging.

#### ECM

The roles of stromal tissue and the ECM in regulating tissue and tumor growth have been long appreciated. In 1889, Paget's proposal (Paget 1889; for review, see Fidler 2003; Oskarsson et al. 2014) that “soil” is as important as “seed” in targeting the growth of cancer metastases first laid out the concept that spatially restricted extratumoral signals may be essential for specifying niches capable of supporting tumor growth. As early as 1911, Peebles’ studies (Peebles 1910) of limb bud engrafting between different sites during chick embryogenesis indicated that the extracellular environment could profoundly influence the fate of tissue differentiation, converting the limb bud fate to that specified by the new environment. Exploration of these observations led to the recognition that the ECM per se could profoundly affect the gene expression and signaling properties of associated cells in a process termed “dynamic reciprocity” (Bissell et al. 1982; Lin and Bissell 1993). This influence of the ECM represents the contribution of both specific proteins present in the tumor microenvironment and also the overall architecture of the tissue matrix and degree of rigidity.

These contributions are highly relevant to all stages of cancer growth. For example, it is well established that mammographic density, which reflects ECM rigidity, is one of the strongest predictors of breast cancer risk (Burton et al. 2017). This density differs between distinct populations, being, for example, higher in Asian and lower in European women. The reasons for these differences are thought to involve height, weight, and parity (Rajaram et al. 2017), although twin studies have indicated at least some genetically heritable component that is not yet well understood (Boyd et al. 2009). However, regardless of the basal density of the mammary tissue, within genetically homogeneous populations, higher breast density is associated with greater cancer risk (Bae and Kim 2016). These relationships clearly imply a role for breast density and ECM rigidity in creating a microenvironment that elevates the risk associated with any tumor-intrinsic initiating mutation. Regional matrix stiffness is significantly increased as tumors begin to grow beyond microscopic precursor lesions, based on a feedback between nascent tumors and surrounding stromal cells that increases tension; these changes promote aggressive growth (Paszek et al. 2005). Some specific somatic mutations that occur in tumors, such as disruptions affecting the TGF-β pathway in pancreatic tumors, act in part by increasing ECM rigidity and fibrosis in the microenvironment, increasing tumor aggressiveness (Laklai et al. 2016). As a further point of complexity, ECM rigidity varies dynamically over the life span, influenced by secreted signals from the senescent cells that accumulate in aging individuals (Lecot et al. 2016) and also by DNA damage response (Rodier et al. 2009). Taken in sum, these studies clearly indicate that cancer risk arising from behavioral or environmental factors likely influences the tissue “soil” as much as the tumor “seed.”

## Is cancer the result of ‘bad luck’? Intrinsic vs. extrinsic causes of human cancers and the role of aging itself in cancer

The idea that cancer risk can be reduced based on modification of behavior or the environment or screening for specific risk-associated mutations is predicated on the idea that these factors are major or predominant contributors to the absolute incidence of cancer. However, an opposing viewpoint is that the major source of cancer risk is biologically programmed and cannot be avoided. This idea is based on the relationship between three key observations. It has long been known that some organ systems are more prone to cancer than others. It has also been long known that some organ systems have a greater regenerative capacity than others, based on increased proliferative potential of individual cells. Finally, cancer risk correlates with age. The significance of the relationship between proliferation rate, organ specificity of cancer risk, and aging has been discussed for more than a century (for review, see Goss 1966; Tomatis 1993). The idea linking these observations is that specific organs undergo replicative replacement throughout the life span of an individual, and this replacement process is marked by an unavoidable error rate, resulting in the gradual acquisition of sets of cancer-promoting mutations (Armitage and Doll 1954; Knudson 1971). Adding relevance to this otherwise philosophical debate is that if a stochastic process is the major driver of cancer risk, the rationale and motivation for devoting significant efforts to prevention are undercut; in contrast, if biological programming is a minor risk factor or can be modified, prevention is strongly justified.

In a highly provocative report, Tomasetti and Vogelstein (2015) used a statistical approach to correlate available information about the stem cell complement of individual tissues in 31 distinct tissue types to estimate the total number of stem cell divisions possible for that organ type and then plotted the results against age of incidence for cancers affecting each of the tissue types for all tumors reported in the U.S. in the Surveillance, Epidemiology, and End Results (SEER) database. This resulted in an extremely high linear correlation of >0.8 by Spearman's *ρ* or Pearson's linear determination (*P* < 5 × 10^−8^), leading the investigators to assert that 65% of the difference in the risk of cancer among distinct tissues related to stem cell divisions over time. They then defined an extra risk score (ERS) as a measurement of overall cancer risk across a lifetime.

Based on these calculations, they estimated that replication rate was sufficient to explain risk for a large number of cancer types that were described as replicative and that prevention was unlikely to be productive for these tumors, which could be ascribed to “bad luck.” In contrast, prevention would be useful for a smaller group of deterministic tumors, where a contribution of environmental or hereditary risk factors could be inferred. This latter group included lung cancer in smokers, cancers associated with HCV or HPV infection, and gastrointestinal tumors associated with hereditary mutations. In a follow-up 2017 study, Tomasetti et al. (2017) extended their work to analyze cancers worldwide using statistical methods and analysis of driver mutations to separate the relative contribution of environmental, replicative, and hereditary effects. Although finding trends similar to those in their earlier analysis, this more comprehensive study led to a more nuanced conclusion, maintaining the emphasis on replicative effects for many tumors but also noting that, for certain tumor types, environment made a major contribution. In sum, the investigators estimated that ∼29% of cancers arise from environmental mutations and are potentially preventable.

The arrival of high-throughput sequencing techniques, making large numbers of cancer genomes available for inspection, allows a reformulation of this debate in molecular terms. Alexandrov and colleagues (Alexandrov et al. 2013; Alexandrov and Stratton 2014) have developed algorithms that assess distinct categories of somatic mutation events to identify underlying signatures that characterized individual tumors. The initial study, analyzing 4,938,362 mutations from 7042 cancers, identified 20 distinct signatures, of which a subset was associated with the age of the patient at cancer diagnosis. A subsequent study focused specifically on these “clock-like” mutational signatures, now analyzing 7,329,860 somatic mutations from 10,250 cancer genomes (Alexandrov et al. 2015). This expanded analysis now identified 33 mutational signatures.

For two of these signatures (nos. 1 and 5), which represented 23% of total mutations detected, the number of mutations increased with age (*P* < 10^−253^) in 26 out of 36 types of cancer assessed. Cancer types marked by this signature included stomach, colorectal, glioblastoma, esophagus, medulloblastoma, and pancreatic, which include several tissue types associated with high replication rates, in accord with the idea that replication-associated defects are particularly relevant in these tumors. Signature 1 appears to be associated with deamination of 5-methylcytosine at CpG dinucleotides, which causes T:G mismatches that are not effectively repaired at replication. In contrast, signature 5 is elevated in other tumor types, including kidney papillary and clear cell cancers and neuroblastoma, and involves C > T and T > C transitions with a transcriptional strand bias, suggesting a possible link to transcription-coupled repair. The investigators hypothesize that the specific elevation of signature 5 in specific kidney tumor types may reflect exposure to a metabolism-associated mutagen abundant in renal tissue, but the mechanism is currently unclear. Both of these age-associated signatures are present at a relatively low level in other common cancers, including breast, melanoma, ovarian, and AML. Furthermore, the fact that even the two aging-associated signatures do not correlate with each other suggests the involvement of some tissue-specific component exclusive of aging.

Importantly, after removal of these two signatures from the overall data set, there was no significant correlation between age and number of mutations, which reflects the remaining 77% of mutations detected (Alexandrov et al. 2015). This suggests that, for these sources, the contribution of replicative effects (replication error) is low and that most of the risk is associated with either hereditary or environmental effects. Although it is likely that further genes associated with hereditary risk will emerge, this pattern suggests a potentially large contribution of environmental factors and a similarly large role for prevention. While it is somewhat difficult to discern the most “important” cancer-inducing mutational source for any given tumor, given the simultaneous presence of multiple signatures (for review, Alexandrov and Stratton 2014), it is clear that there are specific signatures that are associated with specific environmental or behavioral factors, including tobacco smoke (Alexandrov et al. 2016), aristolochic acid (Hoang et al. 2013), and others. Hence, the idea of “bad luck” due to factors such as replication error during cell turnover should be interpreted holistically as one cancer-predisposing element in addition to, or complemented by, preventable procarcinogenic factors.

Another intriguing observation that suggests the importance of modifiable environmental factors is the fact that, even for tumors where there is currently strong evidence for a correlation between abundant stem cell population, high replicative potential, and an age-associated signature of mutations, the pattern of tumor incidence is changing in the general population. For example, while the incidence of colorectal cancer is decreasing overall in the U.S., it is increasing among younger adults, with individuals born in 1990 having twice the risk of colon cancer and four times the risk of rectal cancer as those born in 1950 (Bailey et al. 2015; Siegel et al. 2016, 2017a,b). Such an observation is difficult to explain solely through a stochastic model based on stem cell pools unless one assumes that the size of the stem cell pool is itself affected by factors such as environmental toxins and obesity. This is not inconceivable; a number of proteins that support stem cell self-renewal potential have been shown to be up-regulated and promote aggressive tumor aggressiveness in cancers (e.g., Kudinov et al. 2017) and may have altered expression based on such modifiable factors. Furthermore, as deep genome analysis now begins to address clonality as a critical feature of the emergence of tumors, it is becoming recognized that mutational patterns within a single tumor mass—or even within morphologically normal tissue—can be highly complex, creating uncertainty about absolute mutation rates (Cooper et al. 2015). Deep comprehensive molecular surveys of tumor genomes are an emerging field; ultimately, the ability to compare the mutational spectrum and incidence patterns of tumors diagnosed over multiple decades should definitively inform this debate. At present, the sum of the data available supports the idea of an important role for environmental and behavioral contribution to cancer risk.

While multistep models may explain the dramatic increase in cancer incidence with age, they do not preclude the possibility that complex and potentially reversible aging-related processes might contribute to cancer through systemic changes that favor tumor growth (Campisi 2003, 2013). These aging-related changes may differ from organ to organ and can include diverse processes such as impaired immune response, defects in DNA repair, and altered hormonal environment. As data in support of this idea, mutations that extend life span in mice delay the onset of diseases of old age, including cancer. Furthermore, senescent cells that accumulate with age secrete factors that can be inflammatory, can promote angiogenesis, and can favor the growth of cancer in mouse models (Campisi 2013). In the immune system, the phenomenon of age-related clone hematopoiesis (ARCH), also known as age-related clonal expansion, describes the reduction in clonal diversity among hematopoietic stem and progenitor cells that gradually reduces the functionality of the immune system (Shlush 2018). The molecular basis for ARCH is unclear but is likely to represent a combination of cell-intrinsic mutations and microenvironmental effects; individuals with ARCH are at higher risk for some forms of cancer, including nonhematological cancers (Forsberg et al. 2014). Importantly, the presence of ARCH is correlated with diabetes (Bonnefond et al. 2013), although whether this correlation reflects causation (in either direction) is not yet clear, and ARCH is also correlated with smoking (Coombs et al. 2017). Hence, this aging-related deficiency may be at least partially controllable through prevention methods.

Such observations have stimulated efforts by companies to develop drugs to prevent cancer by delaying aging itself or at least eliminate aged cell populations. Some agents, broadly termed “senolytics” (Zhu et al. 2015), focus on selective removal of senescent cells by various mechanisms; for example, ABT-263/navitoclax, an inhibitor of the anti-apoptotic BCL2 and BCLXL, selectively removes cells that have senesced in response to irradiation or due to normal aging, causing apparent rejuvenation of the hematopoietic system (Chang et al. 2016; Zhu et al. 2016). Similar senolytic effects were seen for other targeted therapies, including dasatinib, HSP90 inhibitors, and other agents (Zhu et al. 2015; Fuhrmann-Stroissnigg et al. 2017).

## The early detection of cancer

Cancer 5-yr survival rates vary substantially between anatomic sites and depend on the size, grade, and stage of the tumor. Stage refers to whether the tumor is local (confined to the organ of origin) or has metastasized regionally (has extended beyond the organ of origin to surrounding tissues or lymph nodes) or distantly (to remote tissues). For all tumors, 5-yr survival rates are best if the tumor is detected at the local stage, although, for some tumors, even early detection is associated with poor survival because of the current lack of effective treatment strategies. For instance, for breast cancer, survival rates are 99% (local), 85% (regional), and 26% (distant) because of excellent therapeutic options, whereas for pancreatic cancer, these rates are 29%, 11%, and 3% (The American Cancer Society 2016, https://www.cancer.org/content/dam/cancer-org/research/cancer-facts-and-statistics/annual-cancer-facts-and-figures/2016/cancer-facts-and-figures-2016.pdf). Thus, a major means of lowering cancer mortality for many cancers is to detect them at the local (or even regional) stage and treat them promptly. For many cancers detected at the local stage, surgical resection may be the only treatment recommended and may be curative.

### Population screening tests for cancer

The chief means of early detection for many cancers is the recognition by the patient that something is awry following the appearance of characteristic signs or symptoms (e.g., the appearance of unusual moles) (Swetter et al. 2016)—hence, campaigns such as the American Cancer Society's “seven warning signs of cancer,” designed to alert patients to visit their doctors and request diagnostic assessment while the cancer is at an early stage. This awareness alone, combined with increased access to health care or the means to pay for it such as Medicare and Medicaid, in the U.S. led to decreases in the proportion of cancers that was detected as distant or late stage, so-called “down-staging.” This is still an underused strategy, particularly in less developed countries (Sankaranarayanan and Boffetta 2010). Other routes to early detection include “opportunistic screening,” in which recommendations for cancer testing are made on a national level but the actual action on the recommendation is up to the individual (the approach used in the U.S.), or “organized screening,” in which testing is systematically offered to asymptomatic high-risk individuals or the general population based on a national program (for example, in Scandinavia).

#### Types of tests

A broad portfolio of evidence-based tests proven to reduce cancer-associated mortality and suitable for application in large populations is currently available to screen for cancer. Additional options ranging from classic medical approaches to new tests based on molecular signatures and other recently established biomarkers are in development. Methods for population testing include fecal occult blood or immunochemical screening or endoscopy of the lower gastrointestinal tract (colorectal cancer), visual inspection of the cervix or cytology (cervical cancer), detection of an oncogenic virus (e.g., HPV for cervical cancer), mammography (breast cancer), radiology (e.g., spiral computed tomography [CT] for lung cancer), or blood-based biomarkers (e.g., PSA [prostate-specific antigen] for prostate cancer) (https://www.uspreventiveservicestaskforce.org/Page/Name/recommendations). These screens fall into two fundamental classes: those that identify premalignant lesions and remove the damaged tissue (e.g., removing adenomas at colonoscopy) and those that indicate that a cancer may be found on further searching and/or biopsy (e.g., fecal occult blood screening). Notably, there are multiple options for some types of cancer, such as breast, colorectal, lung, and cervical cancers, which account for a high proportion of cancers globally. However, there are no feasible options available to screen at the population level for most other types of cancer, highlighting an area where investment in test development might lead to major public health dividends. Also notable is that the equipment and expertise needed to screen varies substantially according to the organ site, and thus screening programs tend to be site-specific, and there are few economies of scale across sites.

#### Technical performance of a screening test

Screening tests are generally evaluated in terms of their sensitivity (the percent of true disease positives, who are called as positive by the screen) and their specificity (the percent of true disease negatives, who are called as negative by the screen). In general, sensitivity needs to be high (e.g., 60%–80%) such that a high proportion of cases is detected by the test. Specificity needs to be even higher (e.g., ≥98%) when the probability of disease is low, as when most cancer screening tests are applied in the general population. This is in part so that the proportion of true negatives (i.e., healthy individuals) who are screen-positive is small, and thus few people suffer the anxiety of a cancer concern and the potential morbidity associated with further diagnostic testing, which is costly and often invasive. Also, at a population level, high specificity is important so that the health system is not overwhelmed by expensive and unjustified follow-up testing (although here the specificity requirement depends on whether the consequences of being labeled positive are relatively minor [e.g., referral to a dermatologist for a skin biopsy] or burdensome [e.g., laparoscopy to diagnose or exclude ovarian cancer]).

Another key performance characteristic is the positive predictive value (PPV); i.e., the proportion of screen positives that are true positives. A good screening test will have a high PPV. An important fact about this metric that is not necessarily intuitively understood is that the PPV of a test varies with the prevalence of the disease being tested for. One way of understanding this is that, at the extremes, the PPV will be zero (if there are no people with the disease in the population tested, then all of the screen positives will be false positives) or 100% (if all people tested have the disease, then all of the screen positives will have the disease). Thus, the PPV varies according to whether the disease is rare or common in the population screened; the rarer the disease is in the population, the higher the fraction of test positives who are false positives will be. This obviously has implications for test development for cancers that are relatively common (e.g., breast, prostate, and lung) versus rare cancers.

#### Programmatic performance of a screening test

A technically excellent screening test is no use if it is (1) too expensive to justify, (2) needs to be repeated too frequently to be feasible, (3) takes too long to generate a result, (4) identifies cancers at such a late stage that treatments are ineffective, (5) or is followed by confirmatory tests that do more harm than good or (6) if people cannot be convinced that they should be screened with the test. Thus, the development of a cancer-screening program involves many actors—the government or insurance companies, the people administering the test and following up the results, and the population being tested being prepared to submit to the test. Because the characteristics of each screening test are different and vary according to the population being screened and the level of development of the health system, no cancer-screening test is universally applied worldwide.

### Early detection methods in the U.S.: underlying mechanisms, successes, limitations, and challenges to delivery

#### Cervical cancer screening

One of the most successful screening tests has been the Papanicolaou (Pap) smear for prevention of cervical cancer. In this test, cells are collected from the opening of the cervix and stained with a mixture of five dyes selected to highlight cellular features, including the keratins found in squamous cell carcinomas. Subsequently, a pathologist evaluates slides to determine whether there is evidence of abnormalities that are characterized as low- or high-grade squamous intraepithelial lesions (LSIL or HSIL, respectively), which represent ∼2%–5%, and <1% of tests, respectively. Cervical cancer mortality in the U.S. has decreased by >50% over the last 40 yr; most of this decline is attributed to the Pap smear (https://www.cancer.org/cancer/cervical-cancer/about/key-statistics.html). By detecting both premalignant and malignant lesions, the Pap smear both reduces the risk of cancer by leading to the removal of premalignant tissues, which are scraped off by colposcopy subsequent to an abnormal test result, and reduces risk of cancer mortality by leading to early diagnosis of curable cancers.

In many less developed countries, however, efforts to introduce the Pap smear have failed, due in part to the time it takes to get an answer (usually the slides are sent to a central laboratory for microscopy, by which time the patient may not be available for follow-up colposcopy). Other issues include the difficulty of getting results back to women who may not have a telephone, postal service, or other means of communication; high costs; the requirement for skilled personnel for interpretation of results; and the lack of personnel for follow-up treatment of positive or suspicious smears (as surgeons and operating theaters may ultimately be needed if a cervical amputation or hysterectomy is required).

Fortunately, developments in molecular biology have enabled advances in cervical cancer prevention and screening in two ways. The finding that oncogenic strains of HPV are a necessary cause of cervical cancer (Walboomers et al. 1999) led to the development of an HPV vaccine that protects women against the majority of the oncogenic HPV strains, including the common HPV16 and HPV18 strains. The two first developed and commercially available vaccines (Gardisil and Cervarix) were virus-like particles (VLPs) based on expression of the major L1 capsid protein derived from multiple oncogenic HPV strains. Initial clinical trials showed 100% efficacy of these vaccines in preventing cervical dysplasia in young women who were HPV-naïve at the time of vaccination 4 yr after administration of a single vaccine dose (for review, see Schiller and Lowy 2012). A current challenge is extending the use of the vaccine, including to young men as well as young women, which is particularly important given the rapidly growing incidence of cancers at noncervical anatomical sites, such as the head and neck (Chaturvedi et al. 2011).

Screening techniques have also improved based on exploitation of the obligate infection of the cervix with high-risk HPV strains prior to detectable Pap abnormalities. HPV testing can entail the use of PCR probes for high-risk HPV strains to amplify viral DNA or a hybrid capture approach to detect the oncogenic strains (Clavel et al. 1998). With respect to screening, several randomized trials have shown that HPV-based screening is superior to Pap/cytology-based screening in protecting against invasive cervical cancer (Ronco et al. 2014; Schiffman et al. 2017).

#### Colonoscopy and other tests for colon cancer

Colorectal cancer is the second leading cause of death in the U.S. (U.S. Preventive Services Task Force 2016) Early detection has a huge impact on preventing death from this disease, and fuller uptake of proven methods of early detection represents an opportunity to realize large gains from population-level screening. Tumors found through screening approaches at an early stage have a 5-yr survival rate of ∼90%; for metastatic colon cancers, the survival rate is 11%. The gold standard method of screening for colon cancer for the past several decades has been by colonoscopy, with a standard recommendation of commencing such testing at age 50 and then testing every 10 yr for members of the general population lacking known risk factors. For individuals with known hereditary risk factors for colorectal cancer (e.g., individuals with Lynch syndrome), similar tests are used but beginning at a much earlier age and with testing performed at frequent intervals (Stoffel et al. 2010). Colonoscopy has been particularly successful because the vast majority of colorectal tumors has a similar life history, progressing through polyps to noninvasive adenomas to adenocarcinomas over many (≥10) years and rarely metastasizing until late stages of tumor growth; concurrent with detection of early stage polyps, these early premalignancies can be readily removed during screening. However, colonoscopy is often avoided due to annoying physical effects associated with clearing the gastrointestinal tract for scoping and due to a lack of access or affordability, with as many as 50% of individuals who would benefit not actually being tested. Alternative approaches, including flexible sigmoidoscopy, CT colonography (CoTCo), the guaiac-based fecal occult blood test (gFOBT), a fecal immunochemical test (FIT; also known as the immunochemical fecal occult blood test [iFOBT], which assesses hemoglobin), and a multitargeted stool DNA test, have become available (U.S. Preventive Services Task Force 2016; Knudsen et al. 2016). Comparative assessments of these tests have indicated that for the general population, colonoscopy every 10 yr, annual FIT, sigmoidoscopy every 10 yr with annual FIT, and CoTCo every 5 yr yielded similar benefits in improving life span if the tests are applied between the ages of 50 and 75. The fact that noninvasive FOBT testing is performing so well suggests that this type of testing may be more readily adopted by individuals unwilling to undergo more invasive screening approaches.

#### Mammography

Although mammography has become established as a means to reduce breast cancer mortality, the data regarding the value of this approach to prevention are surprisingly controversial, with large disagreement on the extent of any reduction in mortality. Most consensus estimates suggest that regular mammography screening reduces breast cancer mortality by ∼20% (Myers et al. 2015). However, this may come at a price of overdiagnosis of breast cancers (i.e., diagnosis of precancerous lesions that would not have been diagnosed as progressive tumors in a woman's lifetime) that some suggest has increased breast cancer incidence by ∼20% (Kalager et al. 2012). The original rationale for mammography at the population level has been the idea that tumor progression goes through a set progression, where tumors reach a minimal size that is detectable by mammography before metastasis occurs. This paradigm has been challenged by a number of recent studies using genomic analysis, analysis of circulating tumor cells (discussed below), and sophisticated imaging techniques to analyze the timing of tumor dispersion. Emerging data suggest that early stage tumors secrete small extracellular vesicles that condition niches to enhance the growth of metastasizing cancer cells (Peinado et al. 2017), with work in mouse models indicating that even very early stage mammary tumors shed circulating tumor cells that can seed such niches (Harper et al. 2016; Hosseini et al. 2016). Similar early dissemination has been seen for other solid tumor types, such as pancreatic tumors (Rhim et al. 2012). Further studies of pancreatic cancer have suggested that such metastases may arise from clonal populations within the larger tumor mass, further uncoupling measurement of tumor size from propensity to metastasize (Yachida et al. 2010). This evolving understanding of metastasis (Massague and Obenauf 2016) supports the idea that prevention approaches focusing on detection and genomic characterization of circulating tumor cells in the peripheral blood may significantly augment mammography in limiting breast cancer mortality (for instance, if effective interception strategies are developed that can control the establishment of metastases before primary tumors are detectable by imaging).

#### PSA testing

A highly contentious screening test is regular testing of men for PSA in order to detect prostate cancer. PSA screening, which became popular in the U.S. and was recommended as an annual test for middle-aged and older men by several expert bodies, was not widely introduced in most European countries. In 2008, the U.S. Preventive Services Task Force recommended against screening men >75 yr and concluded that the evidence was insufficient to the balance of benefits and harms (U.S. Preventive Services Task Force 2008). The results of randomized trials of screening compared with usual care have been much debated, with many men in the nonscreening arm of the major U.S. trial being screened as part of usual care. The most solid conclusions are about the difficulty of doing randomized trials of a screening method that has achieved broad popularity. There is no doubt that PSA testing results in the detection of lesions that pathologists label as cancer in a high proportion of men. Problems with this test include the fact that heterogeneity of prostate cancer aggressiveness among individuals makes it difficult to predict which individuals will develop a life-threatening disease. Many of the lesions detected following a positive PSA test may not have been symptomatic during a man's life, and the follow-up procedures and treatments lead to morbidity (urinary incontinence and/or impotence) in a substantial proportion of treated men. Even if screening results in a net reduction of site-specific cancer mortality, the trade-off against decreased quality of life in treated survivors is a difficult balance for any screening test for which the follow-up confirmation of the diagnosis and/or the treatment of the disease is burdensome.

### A new generation of tests—the ‘liquid biopsy’

Blood-based tests such as PSA screening start with the advantage that the initial screening test does not require expensive equipment (such as spiral CT machines for lung cancer screening), unpleasant preparation (such as the bowel prep for colonoscopy), exposure to radiation (such as mammography), or clinical skills (such as the Pap smear). Thus, there is enthusiasm for the concept that finding tumor markers in the blood may provide a method of routinely screening large populations and potentially replace some of the more burdensome methods (Aravanis et al. 2017). The use of NGS to detect tumor mutations in circulating tumor DNA (ctDNA) that is released by apoptosis or necrosis of tumor cells and/or techniques to detect circulating tumor cells have been shown to identify minimal residual disease and indicate prognosis and treatment response in some types of cancer (Bardelli and Pantel 2017). The sensitivity of these tests may be limited by the fact that most cell-free DNA is derived from normal cells, and the specificity may be limited by the fact that cancer-associated mutations increase with age in tissues that are not yet cancerous (Bardelli and Pantel 2017). Other liquid biopsies focus on the analysis of extracellular vesicles (EVs; comprising exosomes, microvesicles, and oncosomes): small membrane-encased vesicles shed from normal and tumor cells that can transfer nucleic acids and proteins between cells (Strotman and Linder 2016). EVs derived from tumors have attracted great interest, based on evidence that they can perform functions ranging from conditioning the premetastatic niche to immune suppression to control of angiogenesis (Sato and Weaver 2018). In spite of these challenges, there have been some convincing recent studies that tests integrating analysis of tumor proteins and DNA will significantly augment the ability to use “liquid biopsy” to detect early tumors noninvasively and to provide information on the specific mutations present in tumors in cancer patients (Cohen et al. 2017, 2018). Such testing is likely to become routine for some cancers, although the hurdles to converting this information into a test for early detection that could be applied to individuals in the general population remain high. It will be imperative to develop more selective and efficient capture techniques, incorporate advances in bioinformatics and computation, and obtain greater insights and context from cancer biology to further research in this high-priority area of cancer prevention (see also Lewis et al. 2018; Neoh et al. 2018).

## Theoretically preventable U.S. cancers and cancer death

To explore the extent of a maximal impact of prevention through uptake of idealized health-promoting behaviors and policies, we examined two recent studies that quantified these effects (Kohler et al. 2016; Islami et al. 2017). Kohler et al. (2016) summarized modifiable risks from 10 independent large cohorts reported by groups, including the Women's Health Initiative, the National Institutes of Health–American Association of Retired Persons Diet and Health Study, and others. Cumulatively, these cohorts involved >1.5 million individuals and generated a rich data set, including hazard ratios for specific behavioral risk factors identified by the ACS and/or the World Cancer Research Fund/American Institute for Cancer Research (WCRF/AICR). From this information, they were able to estimate resulting preventable cancers and deaths. Islami et al. (2017) modeled the uptake of behavioral risk factors in the U.S. population and related this to cancer incidence and deaths reported in national registries. Notably, the two studies were roughly concordant in their findings, notwithstanding their differing methodologies. This concordance strengthened their conclusions and emphasized the promise of primary prevention in limiting cancer incidence, highlighting the potential of idealized population-wide adherence to recommended healthy behaviors and implementation of policies that reduce cancer risk.

For convenience, we focus on the recent study by Islami et al. (2017) due to their use of population-attributable fractions (PAFs; the proportion of cancer incident cases and deaths that can potentially be prevented due to the elimination of risk factors) as a summary measure. Risk factors were obtained by a meta-analysis of multiple published studies to identify modifiable risk factors for which a causative role in cancer is supported by sufficient or strong evidence. Cancer occurrence and death data were reproduced from Islami et al (2017), who obtained occurrence data from the CDC’s National Program of Cancer Registries (http://www.cdc.gov/cancer/npcr/public-use) and the NCI SEER program (https://www.seer.cancer.gov) and death data from the CDC’s National Center for Health Statistics (https://www.cdc.gov/nchs). Cancer incidence in the U.S. in 2014 was analyzed for 26 cancers for which contributions to incidence from potentially modifiable risk factors (including those discussed in earlier sections) have been demonstrated previously. These data were analyzed in the context of age- and sex-specific risk factor exposures to estimate PAFs; PAFs were then summarized by cancer site and risk factor as well as in aggregate.

Islami et al. (2017) confirmed what has been suggested for years; namely, that a surprisingly large proportion of cancer can be prevented. Through primary prevention alone, they estimated that 45% of incident cancers and 45% of cancer deaths in the U.S. in 2014 were attributable to modifiable risk factors for which they evaluated effects. These factors included tobacco (cigarette smoking and secondhand smoke), “lifestyle” factors (including excess body weight; alcohol; diet, such as red and processed meats and low intake of fruits, vegetables, dietary fiber, and calcium; and physical inactivity), cancer-associated chronic infections (*H. pylori*, HBV, HCV, HPV, HIV [human immunodeficiency virus, which promotes aggressiveness of several virally associated cancers], and HHV8 [human herpesvirus 8, which causes Kaposi's sarcoma]), and UV radiation (from natural and artificial sources).

**Figure 6.**
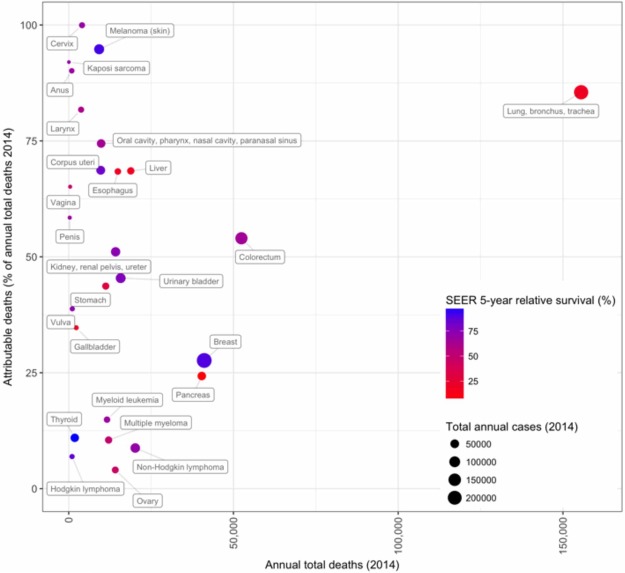
Cancer deaths attributed to modifiable risks. Each anatomically categorized cancer was plotted by the current annual deaths due to that cancer (*X*-axis; total deaths in 2014) and the proportion of deaths attributable (and thus preventable by the elimination of risk factors) to the following modifiable risk factors: tobacco, UV exposure, infections, and Western lifestyle (*Y*-axis; PAF). Circle size is in proportion to cancer-specific incidence (incident cases, 2014), and colors are assigned by SEER 5-yr relative survival estimates (2007–2013; https://seer.cancer.gov/statfacts). Thus, although cancers of the breast and pancreas situate proximally, indicating an approximately equal number of total deaths and a similar PAF for the examined risk factors, breast cancer incidence is much higher (larger point size), and outcomes for breast cancer are far superior (bluish purple in color, indicating a >75% relative survival). Cancers (i.e., lung) shown in the *top right* are those for which we can achieve the greatest reduction in total cancer deaths by the population-wide adoption of healthy behaviors and policies, such as tobacco prevention/cessation or elimination. Cancers shown in the *top left* result in far fewer cancer-associated deaths but may be similarly profoundly reduced through population-wide adoption of healthy behaviors and policies (e.g., avoiding tobacco, cancer-associated infections, and harmful UV exposure). This figure was plotted based on data from Tables 2 and 4 of Islami et al. (2017) and from SEER (https://seer.cancer.gov/statfacts).

Figure 6 is an alternative presentation of findings from Islami et al. (2017) that simultaneously represents the scale of cancers prevented by elimination of modifiable risk factors (“attributable deaths”) and site-specific cancer mortality (annual for the U.S., 2014). Several observations jump out. First, the burden of cancer deaths of the lungs and part of the aero–digestive tract (organs heavily exposed to tobacco in smokers) is exceedingly high even compared with cancer deaths in other common cancer sites, such as the colorectum, breast, and pancreas. Second, these lung cancers reside high in the plot, illustrating that they are largely preventable—at once sobering and motivational. Third, multiple additional sites contributing large numbers of cancer deaths are highly preventable, with >50% attributable to preventable factors for colorectal, kidney, esophageal, and liver cancers as well as melanoma. Finally, cervical cancer is considered to be essentially 100% preventable (via elimination of persistent HPV infection in the population through vaccination).

We note that our presentation, as with Islami et al. (2017), applies to cancers with nontrivial fractions of cases attributable to modifiable risk factors (i.e., primary prevention) rather than screening or early detection (i.e., secondary prevention). Prostate cancer, for example, is omitted. For attributable cancer cases (data not shown), a figure emerges that is similar to that for deaths. If considering only cancer incidence, colorectal and breast are shifted far to the right, with the large number of annual breast cancer diagnoses exceeding even those for lung cancer. The relatively lower mortality for these cancers reflects reductions achieved through treatment, early detection, and screening.

Table 1 presents estimates of attributable cases and deaths by risk factor, with a total attribution from these risk factors estimated to be 660,000 cases (40% of the ∼1.6 million new cancers diagnosed in the U.S. in 2014) and >265,000 deaths (∼45% of the ∼588,000 U.S. cancer deaths in 2014). Tobacco (smoking and secondhand) is the largest contributor to both new cases and deaths, with the cumulative factors associated with an unhealthy lifestyle nearly as impactful for cancer incidence; the greatest contributors to deaths and cases among “lifestyle” factors are obesity (excess body weight; 7.8% PAF) and alcohol (6.5% PAF). The high ratio of cases to deaths for UV exposure is likely due to melanoma being amenable to earlier detection and a resulting relatively favorable survival.

**Table 1.**
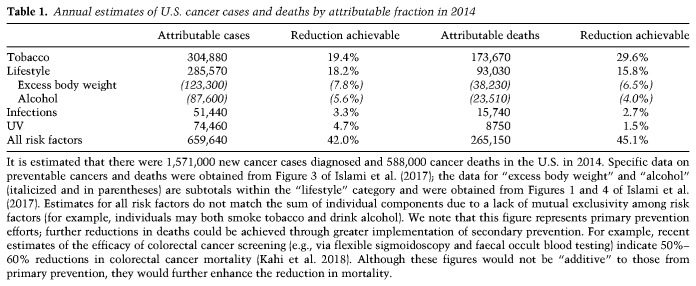
Annual estimates of U.S. cancer cases and deaths by attributable fraction in 2014

There exist caveats to these estimates, as acknowledged by Islami et al. (2017), who wrote the study. Risk factors were assumed to influence cancers independently (not interacting and without correlative effects among cancers). This does not necessarily fully reflect what is known in cancer biology based on emerging evidence about the interaction of risk factors, as summarized above. We also note that the inclusion of only established effects and the incomplete nature of data in cancer registries lead to an underestimate of the total number of cancer deaths attributable to these factors. The stated percentage of “preventable deaths” or cases is thus very much a function of the cancers and risk factors included, the completeness of the surveys, and the population under study, which here is the U.S. population. Due to the specific choice of cancers and risk factors considered in this analysis, the number of deaths that we estimated as avoidable by primary prevention is an underestimate and should thus be considered as a lower bound. For example, prostate cancer is not considered here, but the deaths due to this disease are part of the whole in calculating the 45% figure; certainly, some of these deaths can be attributed to these risk factors or others with modest evidence of causality, some of which are yet to be discovered. We also note that the risk factor exposure estimates were from the most recent year available and thus not averaged over the lifetime of cases, the data for which were also measured in a single time point (i.e., 2014). While Islami et al. (2017) systematically broke out these effects by risk factor and gender and presented measures of uncertainty in their calculations, our goal here was to summarize briefly the theoretical impact of a utopian adoption of practices rather than advocate for any single behavioral change; still, the high potential impact of tobacco prevention/cessation remains stark.

## Conclusion

Since the war on cancer began, there have been continual improvements in survival from many cancers thanks to advances in imaging, surgery, radiation, chemotherapy, and a handful of adjuvant therapies such as tamoxifen. In parallel, there have long been significant efforts to develop resources in education and infrastructure to support screening and prevention behaviors in the general population (Engstrom 1983; Amsel et al. 1987; Fleisher et al. 1988). Today, thanks to stunning research progress that has led to a much better understanding of the biology and genetics of cancer, immunotherapy and some targeted therapies promise additional improvements in treatment outcomes over the next 20–30 yr. Such advances also raise the possibility that, with further research, some cancers might be prevented or treated successfully at very early stages by drugs or vaccines. Despite such success, it is probably fair to say that only by the broader use of proven methods of prevention and early detection, together with treatment, can one guarantee major reductions in current U.S. cancer death rates in the coming two decades.

The fuller uptake of existing methods of prevention and early detection across the U.S. population would also contribute to decreasing disparities in health and longevity that arise from unequal access to the full benefit of these approaches. A meaningful discussion of this topic is beyond the scope of this review. For example, it is now well established in the cancer prevention community that the adverse impact of low SES on prevention is multidimensional and significant. Lower-SES individuals typically have higher rates of depression and anxiety, with attendant higher rates of tobacco and chronic/heavy alcohol use; less access to healthy food/safe environments to permit safe exercise and more obesity; more opportunities for exposure to cancer-associated microbes; and less access to medically based prevention in the form of vaccines, with the attendant lack/delays in evidence-based screening and early detection tests to relevant populations. Therefore, they have more opportunities to be infected with cancer-associated microbes, fail to undergo recommended screening, and experience delays in diagnoses (associated with later stages of disease at presentation), all of which contribute to poor outcomes. For these reasons, effective prevention will require much more than public education on the topic; indeed, most of the potential gains would require actions by governments or large social movements. As a starting point, the interested reader is directed to the following works, and references therein: Colditz and Wei (2012), Stringhini et al. (2017), and Marmot (2018).

However, for the molecular biology community, the topics in this review suggest some areas for productive research. For example, better understanding of critical carcinogenic effects of agents such as obesity, alcohol, and processed meats may lead to the development of targeted interventions that detoxify proximal mutagens and cancer promoters. Better understanding of the mechanism of immune surveillance in cancer may improve cancer vaccines in a manner tailored for individuals with distinct MHC haplotypes. Better understanding of the specific ways in which senescent cells negatively condition the tumor microenvironment may lead to prophylactic interventions that either eliminate specific senescent cell populations or blockade their negative effectors. Importantly, better understanding of how individual genetic variation interacts with specific environmental factors to regulate relative risk is likely to become ever more important as clinical care incorporates “precision” approaches and can help in the development of robust and personalized risk prediction models (Kattan et al. 2016). Microfluidic capture approaches continue to enhance the process of early detection. With an ever clearer view of the mechanistic underpinnings of cancer risk, it becomes more possible to develop tools to counteract these processes, improving quality of life and survival.
